# ERK5 signalling rescues intestinal epithelial turnover and tumour cell proliferation upon ERK1/2 abrogation

**DOI:** 10.1038/ncomms11551

**Published:** 2016-05-17

**Authors:** Petrus R. de Jong, Koji Taniguchi, Alexandra R. Harris, Samuel Bertin, Naoki Takahashi, Jen Duong, Alejandro D. Campos, Garth Powis, Maripat Corr, Michael Karin, Eyal Raz

**Affiliations:** 1 Department of Medicine, University of California, San Diego, La Jolla, California 92093, USA; 2 Sanford Burnham Prebys Medical Discovery Institute, NCI-Designated Cancer Center, La Jolla, California 92037, USA; 3 Laboratory of Gene Regulation and Signal Transduction, Departments of Pharmacology and Pathology, University of California, San Diego, La Jolla, California 92093, USA; 4 Department of Surgery and Science, Graduate School of Medical Sciences, Kyushu University, Fukuoka 812-8582, Japan; 5 Department of Microbiology and Immunology, Keio University School of Medicine, Tokyo 160-8582, Japan; 6 Division of Oral Science for Health Promotion, Niigata University Graduate School of Medical and Dental Sciences, Niigata 951-8514, Japan

## Abstract

The ERK1/2 MAPK signalling module integrates extracellular cues that induce proliferation and differentiation of epithelial lineages, and is an established oncogenic driver, particularly in the intestine. However, the interrelation of the ERK1/2 module relative to other signalling pathways in intestinal epithelial cells and colorectal cancer (CRC) is unclear. Here we show that loss of *Erk1*/*2* in intestinal epithelial cells results in defects in nutrient absorption, epithelial cell migration and secretory cell differentiation. However, intestinal epithelial cell proliferation is not impeded, implying compensatory mechanisms. Genetic deletion of *Erk1/2* or pharmacological targeting of MEK1/2 results in supraphysiological activity of the ERK5 pathway. Furthermore, targeting both pathways causes a more effective suppression of cell proliferation in murine intestinal organoids and human CRC lines. These results suggest that ERK5 provides a common bypass route in intestinal epithelial cells, which rescues cell proliferation upon abrogation of ERK1/2 signalling, with therapeutic implications in CRC.

## Abstract (web summary)

It is unclear how the extracellular signal-regulated kinases 1 and 2 (ERK1/2) pathways interact with other signals in intestinal epithelial cells. Here, the authors show that upon loss of *Erk1/2*, or pharmacological inhibition of MEK1/2, the ERK5 pathway is upregulated to maintain epithelial cell proliferation.

## Graphical abstract

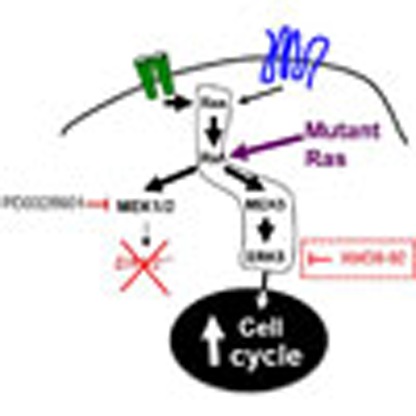

The extracellular signal-regulated kinases 1 and 2 (ERK1/2) are part of the classical family of mammalian mitogen-activated protein kinases (MAPKs), which also include three c-Jun amino-terminal kinases (JNK1/2/3), four p38 isoforms and its lesser-known counterpart, ERK5. The serine/threonine kinases ERK1 (MAPK3, also known as p44 MAPK) and ERK2 (MAPK1, also known as p42 MAPK) show 83% amino acid identity, are ubiquitously expressed and typically activated by growth factors and phorbol esters, whereas the p38 and JNK pathways are mainly activated by inflammatory cytokines and stress^1^. MAPKs are involved in regulation of mitosis, gene expression, cell metabolism, cell motility and apoptosis. ERK1/2 are activated by MEK1 and MEK2, which themselves are activated by Raf-1, A-Raf or B-Raf^1^,^2^. Ras proteins (K-Ras, H-Ras or N-Ras) are small GTPases that can be activated by receptor tyrosine kinases (RTKs) or G-protein coupled receptors (GPCRs), which recruit Raf proteins to the plasma membrane where they are activated. Together, these modules constitute the Ras–Raf–MEK–ERK pathway^3^.

The activation of ERK1/2 results in their nuclear translocation where they can phosphorylate a variety of nuclear targets such as Elk-1, c-Fos and c-Myc^1^, in addition to p90 ribosomal S6 kinases (p90RSKs) and mitogen- and stress-activated protein kinases, MSK1/2. The full repertoire of substrates for ERK1/2 consists of at least 160 cellular proteins^4^. These proteins are typically involved in the regulation of cell proliferation—more specifically, G1/S-phase cell cycle progression—and differentiation. However, their cellular effects are context-dependent and determined by the spatial and temporal dynamics of ERK1/2 activity^5^, which are highly regulated by scaffolding proteins and phosphatases^3^,^6^,^7^.

Despite vast literature on the role of ERK1/2 in cell proliferation, the absolute requirement of this signalling module in rapidly dividing tissues relative to other signalling pathways is unknown. The small intestinal epithelium is particularly suitable to address this question given the short (4–8 days) and dynamic life cycle of intestinal epithelial cells (IECs). Lgr5+ intestinal stem cells at the intestinal crypt base produce transit-amplifying cells, which then undergo a number of proliferative cycles before terminal differentiation into absorptive enterocytes at the crypt–villus border. Enterocytes then migrate to the villus tip where they undergo anoikis and are shed into the gut lumen^8^. All of these cellular events are tightly coordinated by the Wnt, Notch, bone morphogenetic protein (BMP) and Hedgehog pathways^9^, whereas the roles of ERK1/2 remain to be charted. In the intestines, the ERK1/2 pathway is likely activated by autocrine and paracrine factors downstream of RTKs, such as epidermal growth factor receptor (EGFR)^10^, and by exogenous microbial-derived substrates that signal through the Toll-like receptor (TLR)/MyD88 pathway^11^.

To study the effects of ERK1/2 in the adult intestinal epithelium, we generated mice with a conditional (IEC-specific) and tamoxifen-inducible deletion of *Erk2* on the *Erk1*^*−/−*^ background, which completely abrogates this pathway. We show that the ERK1/2 signalling module, surprisingly, is dispensable for IEC proliferation. Genetic deletion of *Erk1/2* in primary IEC or treatment of colorectal cancer (CRC) cell lines with MEK1/2 inhibitors results in compensatory activation of the ERK5 pathway. Moreover, the treatment of human CRC lines with a combination of MEK1/2 and ERK5 inhibitors is more efficacious in the inhibition of cancer cell growth. Thus, compensatory signalling by ERK5 suggests a potential rescue pathway that has clinical implications for targeted therapy in colorectal cancer.

## Results

### Generation of Erk1^*−/−*^ Erk2^fl/fl^ Vil-Cre^ERT2^ mice

**Figure 1:**
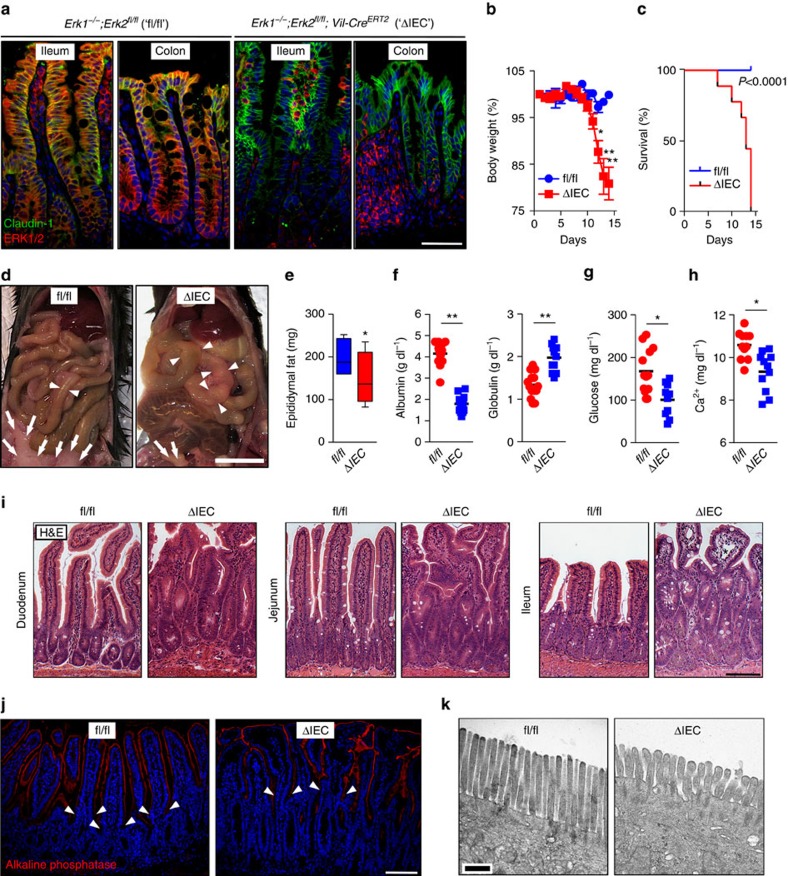
Wasting disease associated with malabsorption in *Erk1/2*^*ΔIEC*^ mice. (**a**) Immunofluorescent staining of paraffin-embedded sections from small intestine or colon taken from *Erk1*^*−/−*^*;Erk2*^*fl/fl*^ (‘fl/fl') and *Erk1*^*−/−*^*;Erk2*^*fl/fl*^*;Vil-Cre*^*ERT2*^ (‘ΔIEC') mice for an IEC marker, Claudin-1 (green) and ERK1/2 (red). Nuclear counter staining with Hoechst (blue). (**b**) Body weight changes of fl/fl and ΔIEC mice (*n*=6 per group) after five consecutive treatments with tamoxifen (1 mg day^−1^). *P<0.05, **P<0.001 by analysis of variance. (**c**) Survival of fl/fl (*n*=10) and ΔIEC mice (*n*=9) after tamoxifen treatment (Kaplan–Meier). (**d**) Macroscopic appearance of the small intestine and abdominal fat pads (indicated by arrows) in fl/fl and ΔIEC mice on day 10 of follow-up. Arrowheads indicate intestinal distension. (**e**) Weight of the epididymal fat of fl/fl and ΔIEC mice (*n*=6 per group) at day 10 of follow-up. (**f**–**h**) Analysis of serum samples from fl/fl and ΔIEC mice. Albumin and globulin levels (**f**), glucose levels (**g**) and calcium levels (**h**) are shown. (**i**) Crypt and villus architecture in the small intestine of fl/fl and ΔIEC mice. Lymphangiectasia marked by (*) was only observed in ΔIEC mice. Haematoxylin and eosin (H&E) staining. (**j**) Alkaline phosphatase (AP) staining of ileum sections from fl/fl and ΔIEC mice. Arrowheads indicate the crypt–villus junction. (**k**) Transmission electron microscopy (TEM) analysis of the brush border of absorptive enterocytes at × 25,000 magnification. Data are presented as mean±s.e.m. (**b**), box-and-whiskers plots (**e**) or scatter plots with mean (**f**–**h**). *P<0.05 and **P<0.001 by Student's *t*-test (**e**–**h**). Scale bars, 1 cm (**d**), 100 μm (**i** and **j**), 50 μm (**a**) or 0.5 μm (**k**).

*Erk1*^*−/−*^ mice are viable and fertile^12^, whereas the *Erk2*^*−/−*^ genotype is associated with embryonic lethality^13^. We generated compound genetically engineered *Erk1*^*−/−*^*;Erk2*^*fl/fl*^*;Vil-Cre*^*ERT2*^ mice by crossing the *Erk1*^*−/−*^;*Erk2*^*fl/fl* 13^ and *Vil-Cre*^*ERT2*^strains^14^. In these mice, treatment with tamoxifen induces IEC-specific deletion of *Erk2* in *Erk1*^*−/−*^*;Erk2*^*fl/fl*^*;Vil-Cre*^*ERT2*^ (referred to hereafter as ‘ΔIEC') but not *Erk1*^*−/−*^*;Erk2*^*fl/fl*^ (‘fl/fl') mice (Supplementary Fig. 1a,c). The specificity of *Erk2* ablation after tamoxifen treatment was demonstrated by genotyping and immunoblotting using lysates of IEC and other tissues (Supplementary Fig. 1b,c). Immunofluorescent staining of small intestine and colon confirmed IEC-specific deletion of ERK1/2; ERK2-positive immunoreactivity in ΔIEC mice was observed only in lamina propria cells and intraepithelial lymphocytes (Fig. 1a). Finally, p-ERK1/2 staining in the small intestine was abundant in the transit-amplifying zone of the crypt, as recently reported^15^ (Supplementary Fig. 1d). This specific staining pattern was only observed in fl/fl mice, whereas p-ERK1/2 immunoreactivity in ΔIEC mice was only detected in lamina propria cells (Supplementary Fig. 1d).

### ERK1/2^ΔIEC^ causes wasting and enterocyte dysfunction

After tamoxifen treatment, ΔIEC mice developed a moribund phenotype between 8 and 10 days of follow-up, whereas their fl/fl littermates remained healthy (Supplementary Fig. 2a). This coincided with the clinical signs of malabsorption, including progressive body weight loss and 100% lethality of ΔIEC mice within 2 weeks of follow-up (Fig. 1b,c). Necropsy was performed at day 10 of follow-up of tamoxifen-treated ΔIEC mice and their fl/fl littermates to evaluate the underlying pathology. ΔIEC mice showed luminal distension of the small intestine and loss of abdominal fat, as well as a trend for shortening of the small intestine and colon (Fig. 1d,e and Supplementary Fig. 2b,c). Serum analysis at day 10 revealed that ΔIEC mice suffered from hypoalbuminaemia, hypoglycaemia, hypocalcaemia and decreased alkaline phosphatase levels when compared with fl/fl littermates (Fig. 1f–h and Supplementary Fig. 2d,e). Together with steatorrhoea (Supplementary Fig. 2f), these findings are indicative of malabsorption. ΔIEC mice also showed lymphocytopenia and increased levels of faecal albumin (Supplementary Fig. 2g,h), consistent with a protein-losing enteropathy. These observations suggest a loss of absorptive surface in the small intestine and/or severe intestinal pathology. Of note, normal serum levels of critical electrolytes, such as Na^+^, K^+^ and PO_4_^3−^, were maintained in ΔIEC mice (Supplementary Fig. 2i). In addition, the serum levels of lipids (triglycerides, cholesterol) were normal or even increased in ΔIEC mice (Supplementary Fig. 2j). This hyperlipidaemia may be associated with loss of gut barrier function^16^, which also occurred in ΔIEC mice (Supplementary Fig. 2h,k), leading to lipid accumulation in the intestinal lamina propria (Supplementary Fig. 3a). Since the *Vil-Cre*^*ERT2*^ model may lead to recombinase activity in the kidney^14^, which could explain some of the observed metabolic aberrations, we analysed kidney function in fl/fl and ΔIEC mice. We did not observe any microscopic differences in the parenchymal tissue indicative of kidney failure, nor increased blood urea nitrogen levels in ΔIEC mice (Supplementary Fig. 2l,m), suggesting the observed phenotype was primarily caused by intestinal pathology.

Histological analysis showed that the crypt–villus morphology was severely perturbed in ΔIEC mice. This included signs of crypt elongation and villus shortening, more severely in the ileum compared with the jejunum and duodenum, respectively (Fig. 1i). Notably, at the most severely affected sites in the ileum, there was dilation of lacteals in villi, also known as intestinal lymphangiectasia (Fig. 1i and Supplementary Fig. 3b), consistent with protein-losing enteropathy. Even though alkaline phosphatase (AP) staining did not show differences by confocal fluorescence microscopy (Fig. 1j and Supplementary Fig. 3c), detailed analysis of the brush border on enterocytes by electron microscopy revealed that microvilli of ΔIEC mice were short, thick and non-uniform (Fig. 1k), a hallmark of defective enterocyte differentiation. Together, these observations indicate a malabsorption and protein-losing enteropathy phenotype, resulting in rapid lethality of ΔIEC mice. These data suggest an essential role for the ERK1/2 signalling module in the orchestration of normal crypt–villus architecture, maintenance of the gut barrier and absorptive enterocyte maturation in the small intestine.

### ERK1/2^ΔIEC^ has no major effects on colon homeostasis

Although efficient knockout was achieved in the colon by tamoxifen treatment in *Erk1*^*−/−*^*;Erk2*^*fl/fl*^*;Vil-Cre*^*ERT2*^ mice (Fig. 1a and Supplementary Fig. 1b,c), no gross alterations were observed in the colon crypt architecture resulting from ΔIEC (Supplementary Fig. 3d). More detailed analysis showed a loss of the luminal, flat epithelial sheet in the colon (Supplementary Fig. 3d–g), analogous to the villous changes in the small intestine. However, *Erk1/2* deletion in the colon did not result in perturbation of goblet cell differentiation or an increased rate of apoptosis (Supplementary Fig. 3h–k). Given the lack of a spontaneous phenotype in the colon in ΔIEC mice, we focused on the changes in physiology in the small intestine hereafter.

### ERK1/2^ΔIEC^ leads to migration and differentiation defects

**Figure 2:**
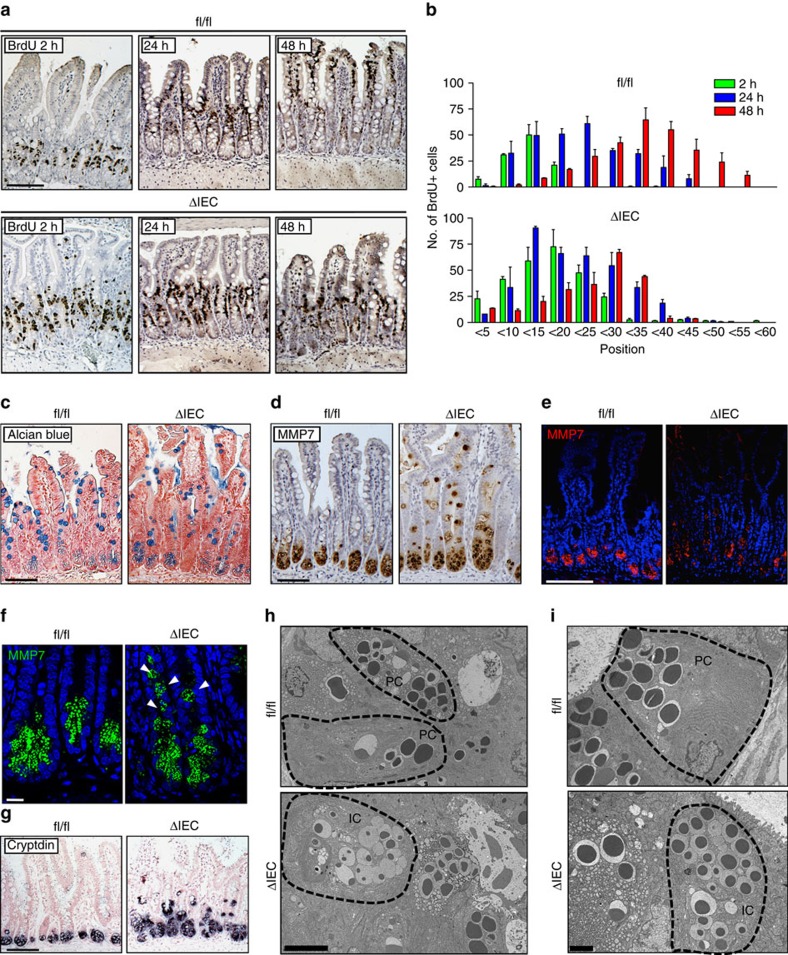
Abnormal IEC migration and secretory cell differentiation in *Erk1/2*^*ΔIEC*^ mice. (**a**–**g**) Comparison of ileum sections from fl/fl and ΔIEC mice 10 days after initiation of tamoxifen treatment. Representative examples from BrdU pulse-chase labelling experiments (2, 24 and 48 h) with ileum sections (**a**). Small intestinal sections without the aforementioned typical villous atrophy observed in ΔIEC mice were selected for the analysis. The position of BrdU-positive cells along the crypt–villus axis at 2 (green), 24 (blue) or 48 h (red) after injection (**b**). Results are mean±s.e.m. of 10 crypts (*n*=2 per group). Immunohistochemical staining for Alcian blue (**c**) and MMP7 (**d**). Immunofluorescent staining for MMP7 at low magnification to show crypts and villi (**e**), and at high magnification to show MMP7+ staining cells at the crypt base (**f**). Detection of *Cryptdin* transcripts by *in situ* hybridization; counter staining with nuclear red (**g**). (**h**,**i**), Ileum sections from fl/fl and ΔIEC mice 10 days after tamoxifen treatment were visualized by transmission electron microscopy (TEM). Ultrastructural features of secretory epithelial cells at the crypt base at × 2,500 magnification (**h**). Higher magnification of Paneth cells in the crypt at × 6300 magnification (**i**). IC, intermediate cell; PC, Paneth cell. Scale bars, 100 μm (**a**,**c**–**e**, **g**), 10 μm (**f**), 5 μm (**h**) or 2 μm (**i**).

We hypothesized that the architectural derangement in the small intestine of ΔIEC mice might be explained by a migration defect on loss of epithelial ERK1/2 signalling. Consistent with this, we observed retention of AP-positive IECs in small intestinal crypts in ΔIEC mice, whereas AP immunoreactivity is normally not observed distal to the crypt–villus junction (Fig. 1j and Supplementary Fig. 4a). BrdU pulse-chase experiments demonstrated a dramatic reduction in the migratory rate of epithelial cells in ΔIEC mice compared with fl/fl littermates (Fig. 2a,b). This defect appeared to involve mostly migration in the villous compartment, which was observed >24 h after BrdU labelling. Differentiation and maturation of epithelial cells from the secretory lineage—most prominently, goblet and Paneth cells—are directly correlated with their position along the crypt–villus axis. The long-lived Paneth cells make antimicrobial peptides and provide a stem cell niche at the crypt base; goblet cells produce mucus^17^. Less abundant secretory cell types include enteroendocrine cells that release a variety of hormones, and Tuft cells that produce eicosanoids^17^. These secretory cells, with the exception of Paneth cells, follow the migration pattern of absorptive enterocytes and share their fate at the villus tip. Indeed, we observed reduced numbers and an irregular distribution of Alcian blue-positive staining goblet cells in the small intestine of ΔIEC mice when compared with fl/fl littermates (Fig. 2c, Supplementary Fig. 4b–d). Paneth cells, which can be identified by positive staining for the granule protein matrilysin (also known as matrix metalloproteinase 7, MMP7), displayed more dramatic changes. Paneth cells in ΔIEC mice were mislocalized along the crypt–villus axis, showed reduced granule contents and had smaller secretory granules that were heterogeneous in size and found outside the apical cytoplasm (Fig. 2d–f). MMP7+ cells in the small intestine of ΔIEC mice also showed a more rounded morphology rather than their regular wedge-like shape in fl/fl control littermates, even though they expressed the prototypical Paneth cell marker *Cryptdin* (Fig. 2f,g). Alcian blue/MMP7 double staining showed an increased number of cells expressing the Paneth cell marker, with a relative loss of mucin positive cells (Supplementary Fig. 4c,d). We hypothesized that these observations might be explained by a differentiation block in the secretory lineage, resulting in the accumulation of immature ‘intermediate' cells—a precursor cell type that is normally rarely found in the lateral wall of the crypt base^18^. We did not observe any intermediate cells in the ileal sections from fl/fl mice, which showed the typical electron microscopic features of mature goblet and Paneth cells, respectively (Fig. 2h,i and Supplementary Fig. 4e). In contrast, intermediate cells were evident in the ileal sections from ΔIEC littermates (Fig. 2h,i), with their goblet cells showing larger electrodense cores, and conversely, Paneth cells displaying smaller cores and increased amounts of free ribosomes in the cytoplasm (Supplementary Fig. 4f). These data suggest that ERK1/2 signalling is critical for the migration of IEC along the crypt–villus axis, in addition to the full differentiation and maturation of IEC from the secretory cell lineage.

### Abrogation of ERK1/2 results in enhanced ERK5 signalling

**Figure 3:**
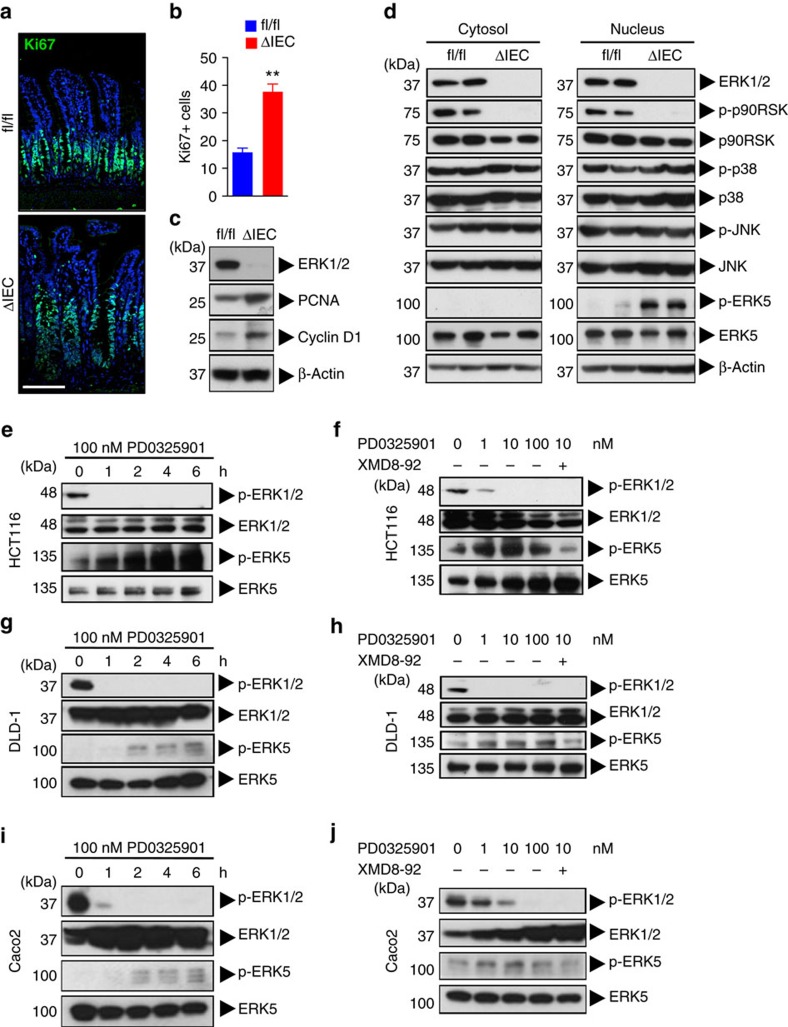
Abrogation of ERK1/2^IEC^ results in enhanced ERK5 signalling. (**a**,**b**) Ki67 staining of paraffin-embedded ileum sections from fl/fl and ΔIEC mice, 10 days after tamoxifen treatment. Nuclear staining with Hoechst. Sections were analysed by confocal fluorescent imaging; scale bar, 100 μm (**a**). Enumeration of the number of Ki67-positive cells per crypt (**b**). Data are mean±s.e.m. of 10 crypts (*n*=3 per group). **P<0.001 (Student's *t*-test). (**c**) Immunoblotting for indicated proteins with IEC lysates isolated from fl/fl and ΔIEC mice as in **a**. (**d**) Immunoblotting for indicated proteins with IEC lysates as in **a**. (**e**) HCT116 cells were treated with the specific MEK1/2 inhibitor, PD0325901, for 0, 1, 2, 4 or 6 h and analysed by immunoblotting for indicated proteins. (**f**) HCT116 cells were treated with PD0325901 (0, 1, 10 and 100 nM) for 6 h and analysed by immunoblotting for indicated proteins. As control, some cells were co-treated with the specific ERK5 inhibitor, XMD8-92 (10 μM). (**g**) DLD-1 cells were treated as in **e**, and cell lysates analysed by immunoblotting. (**h**) DLD-1 cells were treated as in **f**, and lysates were analysed by immunoblotting. (**i**) Caco2 cells were treated as in **e**, and lysates were analysed by immunoblotting. (**j**) Caco2 cells were treated as in **f**, and analysed by immunoblotting. All data are representative of two to three experiments.

Despite the well-established role of MAPK ERK1/2 signalling in cell cycle progression, there were no signs of epithelial cell hypoproliferation in ΔIEC mice. In fact, we observed crypt hyperplasia and an increased number of Ki67+ cells in the ileum (Fig. 3a,b and Supplementary Fig. 5a) and to a lesser degree in the colon (Supplementary Fig. 5b,c) of ΔIEC mice compared with fl/fl littermates. This was associated with increased expression of the proliferation marker PCNA and cell cycle-associated protein cyclin D1 (Fig. 3c). Upregulation of cyclin D1 promotes progression through the G_1_/S-phase, thus indicating that on genetic ablation of ERK1/2, cell cycling in the intestinal epithelium remains intact. There was no direct evidence for upregulated Wnt/β-catenin signalling in IEC after ERK1/2 deletion *in vivo* (Supplementary Fig. 5d), or after siRNA-mediated knockdown in TOPflash reporter cell lines *in vitro* (Supplementary Fig. 5e–g). We hypothesized that an alternative MAPK pathway may compensate for the loss of ERK1/2 signalling to drive the hyperproliferative phenotype of ΔIEC mice. We did not observe compensatory upregulation of p38 or JNK phosphorylation upon ERK1/2 abrogation (Fig. 3d). In contrast, constitutive levels of phospho-ERK5 were undetectable in fl/fl mice, whereas ERK1/2 abrogation resulted in a marked increase of ERK5 phosphorylation (Fig. 3d). Loss of constitutive phosphorylation of p90RSK confirmed sustained abrogation of signalling downstream of the ERK1/2 module in ΔIEC mice, despite the concomitant ERK5 activation (Fig. 3d).

To further study the interactions between the ERK1/2 and ERK5 pathways, we used human colorectal cancer (CRC) cell lines HCT116 and DLD-1 that harbour heterozygous *KRAS*^*G13D*^ (gain-of-function) mutations. Treatment of HCT116 cells with the specific MEK1/2 inhibitor, PD0325901, resulted in the upregulation of p-ERK5 levels in a time- and dose-dependent manner (Fig. 3e,f), suggesting compensatory ERK5 activation upon inhibition of the ERK1/2 pathway. Specificity of p-ERK5 immunoblotting was demonstrated by reversal of the signal by co-treatment with an ERK5 inhibitor, XMD8-92 (Fig. 3f). Similar results were achieved with the DLD-1 cell line (Fig. 3g,h), as well as Caco2 cells (Fig. 3i,j). The latter cell line is *KRAS* wildtype, which suggests that upregulation of ERK5 signalling occurs independently of *KRAS* mutation status. These data are consistent with the upregulated p-ERK5 levels we observed in ΔIEC mice that do not harbour oncogenic mutations. Since abrogation of the Raf–MEK–ERK module can result in feedback activation of EGFR^19^, which could then be responsible for supraphysiological activity of the ERK5 pathway^20^,^21^, we tested whether EGFR inhibition prevented ERK5 upregulation. However, treatment of DLD-1 or Caco2 cells with MEK1/2 inhibitor in the presence of the EGFR inhibitor, erlotinib, still resulted in enhanced p-ERK5 levels (Supplementary Fig. 5h). These data suggest that the compensatory upregulation of the ERK5 pathway upon abrogation of ERK1/2 signalling takes place through an EGFR-independent mechanism.

### ERK1/2 and ERK5 are interconnected signalling pathways

**Figure 4:**
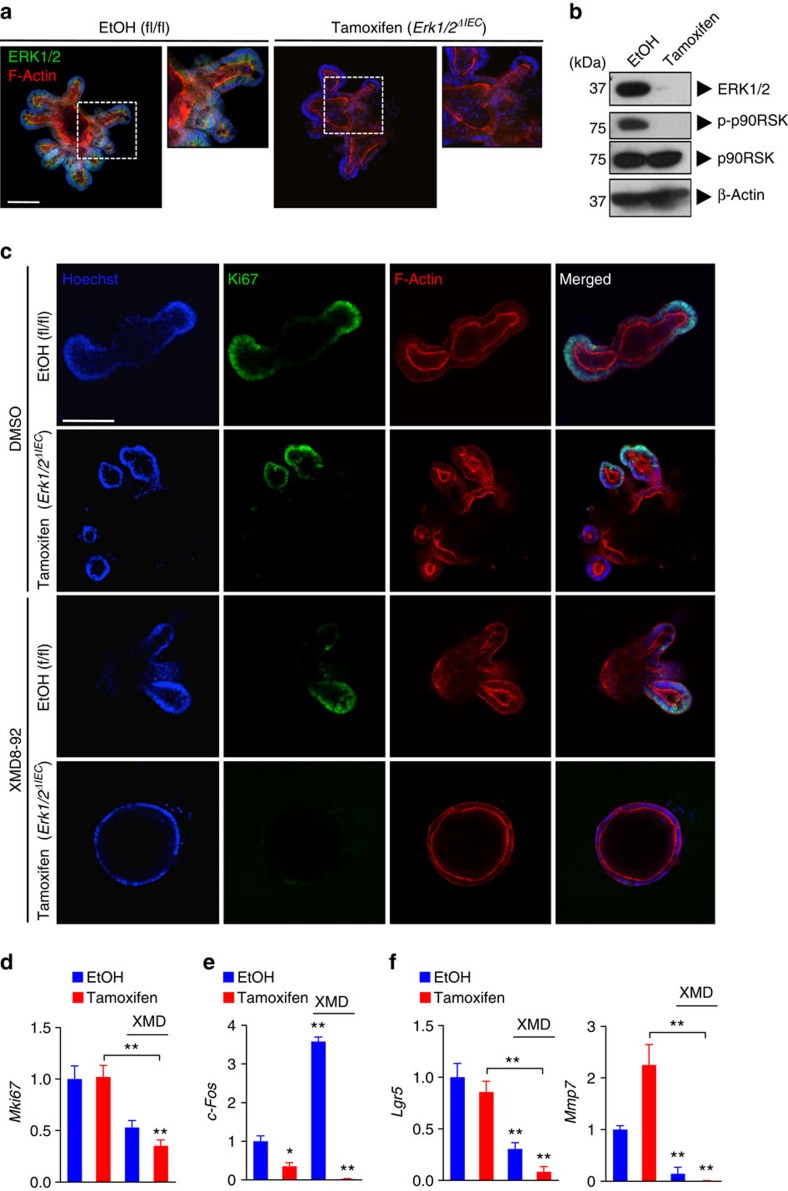
ERK5 signalling is required for the proliferation of *Erk1/2*^ΔIEC^ organoids. (**a**,**b**) Small intestinal organoids generated from naive ΔIEC mice were treated with EtOH or tamoxifen (0.5 μM) for two consecutive days. Organoids were stained for ERK1/2 with counter staining for F-actin (phalloidin-AF546), on day 5 after initiation of treatment, and analysed by confocal fluorescent imaging (**a**). Immunoblotting with intestinal organoid lysates for indicated proteins (**b**). (**c**–**f**) Intestinal organoids were treated with EtOH or tamoxifen (0.5 μM) in combination with DMSO or XMD8-92 (10 μM), respectively, and analysed on day 5. Ki67 staining and confocal fluorescent imaging (**c**). Analysis of RNA samples by Q-PCR (*n*=2–3 per group) for *Mki67* (**d**), *c-Fos* (**e**), *Lgr5* and *Mmp7* (**f**), normalized to *Gapdh*. Data are mean±s.e.m. *P<0.05, **P<0.01 versus control (EtOH) or as indicated, by analysis of variance. Scale bars, 100 μm (**a** and **c**). All data are representative of at least two independent experiments.

We addressed the role of the ERK1/2 versus ERK5 signalling modules on cell proliferation in primary IEC cultures. To exclude any indirect effects by stromal, hematopoietic or neuronal cells on IECs, we used the small intestinal organoid culture system^22^. Intestinal organoids were generated from untreated ΔIEC mice and, after purification and several passages, treated with tamoxifen or its vehicle (ethanol) for two consecutive days *in vitro*. This protocol resulted in rapid and robust (>98%) knockout of *Erk2* transcripts in tamoxifen-treated organoids (Fig. 4a,b and Supplementary Fig. 6a–c). Genetic ablation of *Erk1/2* did not interfere with crypt budding or cell proliferation in intestinal organoids, analogous to our observations *in vivo*, but resulted in morphological changes (Supplementary Fig. 6a,d), possibly related to migration and differentiation defects. We evaluated the requirement for ERK5 signalling in *Erk1/2*^*ΔIEC*^ organoids for cell proliferation. Treatment with tamoxifen or with ERK5 inhibitor, XMD8-92, alone did not prevent organoid growth, whereas co-treatment resulted in the disappearance of budding crypts and a marked loss of expression of proliferation marker Ki67 (Fig. 4c,d and Supplementary Fig. 6f). Genetic deletion of *Erk2* was confirmed by quantitative PCR (Q-PCR) analysis (Supplementary Fig. 6e). Expression of the ERK1/2 target gene *c-Fos* was abrogated by tamoxifen treatment but increased upon ERK5 inhibition, suggesting compensatory upregulation; this was abolished upon co-treatment with XMD8-92 (Fig. 4e). Furthermore, targeting ERK1/2 and ERK5 together inhibited expression of the intestinal stem cell marker, *Lgr5*, and the Paneth cell marker, *Mmp7* (Fig. 4f). Both the cell types are crucial for the regenerative potential of the intestinal crypt. These data suggest that ERK1/2 and ERK5 act as interconnected signalling pathways that maintain the self-renewal capacity of the intestinal epithelium.

**Figure 5:**
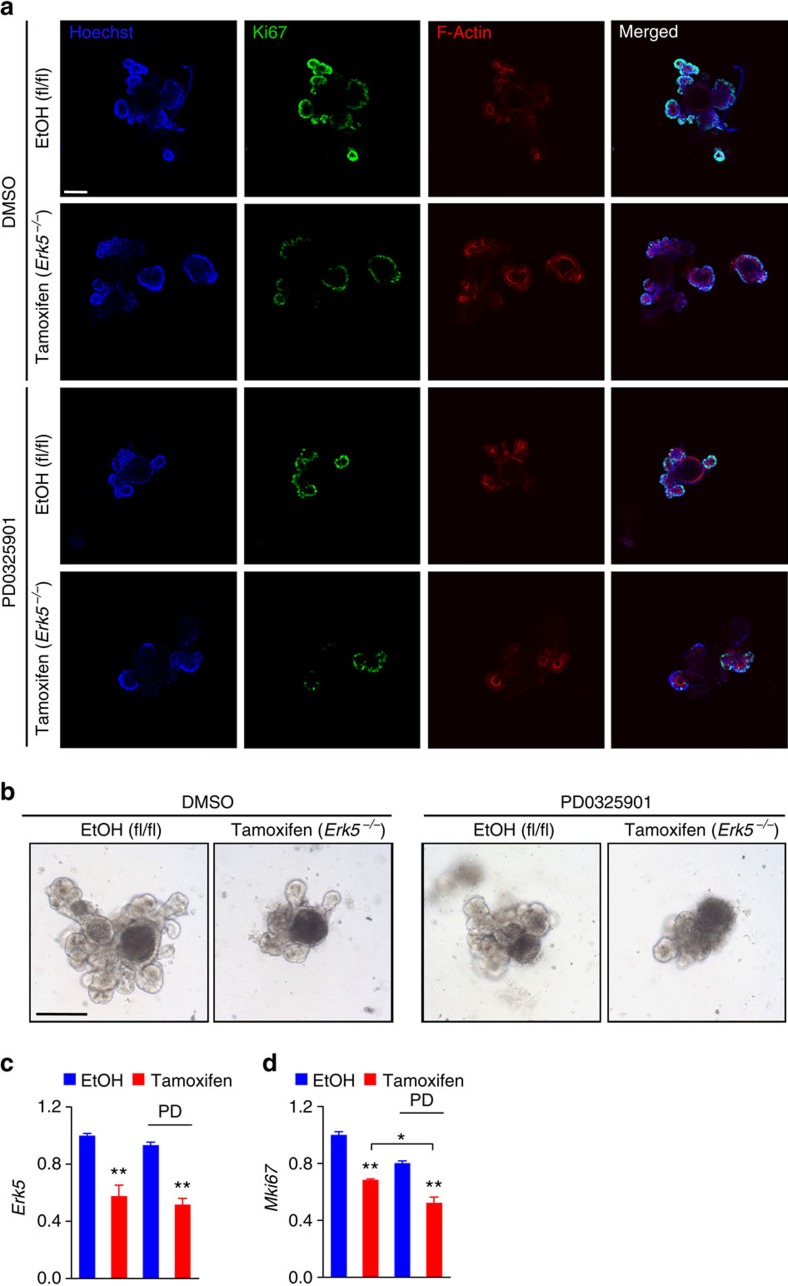
ERK1/2 signalling is required for *Erk5*^*−/−*^ intestinal organoid growth. Intestinal organoids were generated from *Erk5*^*fl/fl*^ mice and transduced with Cre^ERT2^ pMSCV retrovirus. Organoids were then treated with EtOH or 4-hydroxytamoxifen (0.5 μM), together with DMSO or PD0325901 (100 nM) for 2 days. Organoids were analysed on day 5 of culture and imaged by confocal microscopy after immunofluorescent staining for Ki67 and F-actin (**a**). Morphology of EtOH or tamoxifen treated *Erk5*^*fl/fl*^ organoids after transduction with Cre^ERT2^ retrovirus, with or without co-treatment with PD0325901, visualized by live-cell brightfield microscopy (**b**). Analysis of *Erk5* (**c**), and *Mki67* (**d**) transcript levels in organoids after treatment, measured by Q-PCR and normalized to 18 S rRNA (*n*=2–3 per group). Data are mean±s.e.m. *P<0.05, **P<0.01 versus control (EtOH) or as indicated, by analysis of variance. Data are representative of at least two experiments. Scale bars, 100 μm (**a** and **b**).

To confirm these findings in IEC with genetic deletion of *Erk5*, intestinal organoids were generated from *Erk5*^*fl/fl*^ mice^23^,^24^. Established organoid lines were then transduced with retroviral Puro-Cre^ERT2^ or lentiviral Puro-Cre, followed by puromycin selection. Tamoxifen treatment of these transduced intestinal organoids resulted in ∼50% reduction of *Erk5* mRNA levels (Fig. 5c). The deletion of *Erk5* did not result in enhanced levels of p-ERK1/2 in intestinal organoids cultures (Supplementary Fig. 6g). In accordance with the aforementioned data, we found that tamoxifen-induced reduction of *Erk5* expression in combination with PD0325901 treatment was more efficient in abrogating cell proliferation when compared with either condition alone (Fig. 5a–d).

### Targeting ERK1/2 and ERK5 inhibits tumour cell proliferation

**Figure 6:**
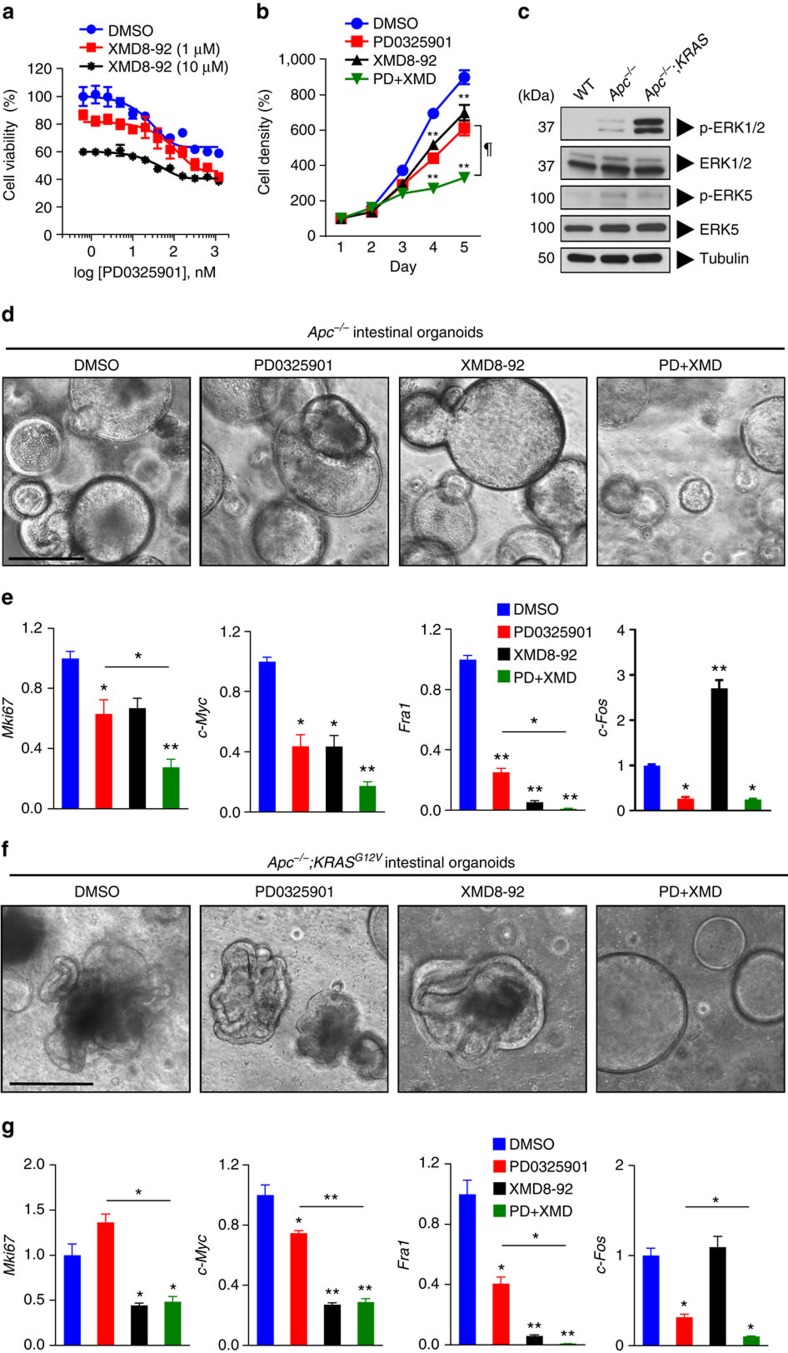
Co-targeting MEK1/2 and ERK5 pathways inhibits tumour cell growth. (**a**) HCT116 cells were plated at 5 × 10^3^ cells per well and treated with PD0325901 (0.63, 1.25, 2.5, 5.0, 10, 20, 40, 80, 160, 320, 640 or 1280, nM) with or without XMD8-92 (0, 1 or 10 μM). Cell viability was assessed by XTT assay after 48 h of treatment. (**b**) HCT116 cells were plated at 2 × 10^3^ cells per well and cultured in the presence of DMSO, PD0325901 (100 nM), XMD8-92 (10 μM) or PD0325901 (100 nM)+XMD8-92 (10 μM). Compounds were added on day 0 and day 2. XTT assay was performed on five consecutive days. Results are normalized to cell densities on day 1 (*n*=8 per condition). (**c**) Small intestinal organoids were generated from WT or *Apc*^*−/−*^ mice (first two lanes). Some *Apc*^*−/−*^ organoids were transduced with *KRAS*^*G12V*^ lentivirus (third lane). Organoids were then analysed for the indicated signal transducers by western blotting. (**d**,**e**) Intestinal organoids generated from *Apc*^*−/−*^ mice were treated with DMSO, PD0325901 (20 nM), XMD8-92 (10 μM) or PD0325901 (20 nM)+XMD8-92 (10 μM) for 5 days. Organoids were visualized by live-cell brightfield microscopy at the end of treatment (**d**), and analysed by Q-PCR (*n*=3 per condition) for expression of *Mki67*, *c-Myc*, *Fra1* and *c-Fos* transcripts, normalized to *Gapdh* (**e**). (**f**,**g**) Intestinal organoids generated from *Apc*^*−/−*^ mice were transduced with *KRAS*^*G12V*^ lentivirus, and treated with MAPK inhibitors as in **d**. The resultant *Apc*^*−/−*^;*KRAS*^*G12V*^ organoids were analysed by live-cell brightfield microscopy (**f**) and Q-PCR (*n*=3 per condition) for expression of *Mki67*, *c-Myc*, *Fra1* and *c-Fos* transcripts, normalized to *Gapdh* (**g**). All data are mean±s.e.m. *P<0.05, **P<0.001 versus DMSO, ^¶^*P*<0.01 for PD0325901 versus PD+XMD (**b**), *P<0.05, **P<0.001 versus DMSO or as indicated, by analysis of variance (**e**,**g**). Scale bars, 100 μm (**d** and **f**).

Given the functional interconnection between the ERK1/2 and ERK5 pathways in IEC, we next evaluated the effects of targeted inhibition of both signalling modules on tumour cell proliferation. In a two-dimensional growth model, we found that treatment of the colorectal cancer cell line, HCT116, with the MEK1/2 inhibitor, PD0325901, was more effective in suppressing cell proliferation in combination with ERK5 inhibitor, XMD8-92, compared with either treatment alone (Fig. 6a,b). Similar results were achieved by measuring cells in S/G2/M-phase by propidium iodide labelling after treatment with DMSO, MEK1/2 inhibitor alone, ERK5 inhibitor alone or the combination of both inhibitors, respectively (Supplementary Fig. 7a,b). These results were confirmed in the other CRC cell lines, including DLD-1, HT-29 and SW480 (Supplementary Fig. 7c,d). Of note, siRNA-mediated knockdown of ERK1/2 and/or ERK5 validated these MAPKs as drug targets in CRC, although cell lines were relatively more susceptible to ERK1/2 knockdown (Supplementary Fig. 7e,f). We then used a three-dimensional growth model with intestinal organoids generated from *Apc*^*fl/fl*^ mice^25^, that were transduced with Adeno-Cre *in vitro* (referred to hereafter as ‘*Apc*^*−/−*^'). Intestinal organoids with a somatic deletion of tumour suppressor APC display upregulated Wnt signalling activity, which results in a rounded, cyst-like morphology, distinct from wild-type organoids^26^ (Supplementary Fig. 7g). We have previously shown that ERK1/2 signalling is required for Wnt-driven adenoma formation *in vivo*^11^. Furthermore, intestinal organoids that are APC deficient showed upregulated levels of p-ERK1/2 and p-ERK5 compared with WT organoids (Fig. 6c). In line with this, PD0325901 treatment inhibited organoid growth and the expression of proliferation marker *Mki67* and oncogenes *c-Myc* and *Fra1*, which was also observed with XMD8-92 treatment, with the combination treatment being superior to either treatment alone (Fig. 6d,e). Synergistic, specific or antagonistic effects of the MAPK inhibitors were observed on the expression of stem cell markers *Lgr5* and *Ascl2*, Paneth cell marker *Mmp7* and immediate-early gene *Egr1*, respectively (Supplementary Fig. 7h). Confirmation of on-target effects of the inhibitors was confirmed by the analysis of *c-Fos* expression and immunoblotting for MAPK proteins (Fig. 6e, Supplementary Fig. 7i). Notably, we observed that ERK5 protein levels were increased on long-term MEK1/2 inhibitor treatment, whereas ERK5 inhibitor treatment resulted in reduced ERK5 protein levels (Supplementary Fig. 7i), suggesting antagonistic interactions between the two MAPK modules. Suppression of total ERK5 levels by long-term treatment with XMD8-92 was also observed in multiple CRC lines (Supplementary Fig. 7j). Together, these data suggest that ERK1/2 and ERK5 are major, interrelated transducers of extracellular mitogenic signals.

Next, we assessed the effects of MAPK inhibitors on the growth of *Apc*^*−/−*^ organoids that express mutant *KRAS*^*G12V*^ by lentiviral transduction. These organoids showed marked morphological differences compared with both wild-type and *Apc*^*−/−*^ organoids (Supplementary Fig. 7g), suggesting abnormal cellular homeostasis, reminiscent of oncogenic KRAS signalling in human CRC. Mutant *KRAS* expression appeared to preferentially activate ERK1/2 compared with ERK5 in organoids on the *Apc*^*−/−*^ background (Fig. 6c). Treatment with MAPK inhibitors showed that only the combination of MEK1/2 and ERK5 inhibitors reversed the typical morphological changes induced by oncogenic KRAS on the *Apc*^*−/−*^ background (Fig. 6f). Interestingly, treatment with ERK5 inhibitor was superior over MEK1/2 inhibitor with regard to suppression of proliferation and expression of the oncogene *c-Myc*, stem cell markers *Lgr5* and *Ascl2* and Paneth cell marker *Mmp7* (Fig. 6g, Supplementary Fig. 7k), suggesting differential effects of abrogating MEK1/2 versus ERK5 signalling depending on KRAS activity in three-dimensional growth. The direct effects of MEK1/2 and ERK5 inhibition were validated by the evaluation of *c-Fos* expression and immunoblotting for MAPK proteins (Fig. 6g, Supplementary Fig. 7l). Thus, these data suggest that pharmacological inhibitors of ERK5 can be exploited to target Wnt-driven intestinal tumour cell proliferation, and may be superior over MEK1/2 inhibitors in CRC tumours depending on KRAS mutation status.

## Discussion

Here we show that ERK1/2 signalling in mouse intestinal epithelium is dispensable for cell proliferation, while it resulted in abnormal differentiation of enterocytes, wasting disease and ultimately lethality (Fig. 1). Consistent with these findings, ERK1/2 MAPKs were shown to be associated with the enterocyte brush border and activated upon RTK stimulation or feeding^27^ or electrical field stimulation in polarized epithelium^28^. This seems at odds with literature that suggest that maintained ERK1/2 signalling precludes enterocyte differentiation^29^,^30^. A possible explanation for this discrepancy could be that cycling IEC in the transit amplifying zone of the crypt require relatively high levels of active ERK1/2, which is readily blocked by pharmacological intervention, whereas a transition to low level ERK1/2 activity in IEC migrating into the villus compartment promotes the absorptive enterocyte differentiation program that is only perturbed upon complete genetic deletion of *Erk1/2.* Little is known about the role of ERK1/2 signalling in the life cycle of secretory cells in the gut. A recent report by Heuberger *et al*.^15^ described that IEC-specific deletion of non-receptor tyrosine phosphatase, Shp2, resulted in the loss of p-ERK1/2 levels in the small intestine. This coincided with an increased number of Paneth cells at the expense of goblet cells in the small intestine, as well as shortening of villi. They also observed the strongest staining for epithelial p-ERK1/2 in the TA zone. This p-ERK1/2+ staining pattern and the architectural organization of the TA zone were lost in *Shp2* knockout mice. Interestingly, the deleterious effects of *Shp2* deficiency were rescued by expression of constitutively active MEK1. A model was proposed in which the balance between Wnt/β-catenin and MAPK signalling determines Paneth cell versus goblet cell differentiation, respectively^15^. This proposed crucial role for ERK1/2 MAPK signalling in intestinal secretory cell differentiation is consistent with our observations in *ERK1/2*^Δ*IEC*^mice.

**Figure 7:**
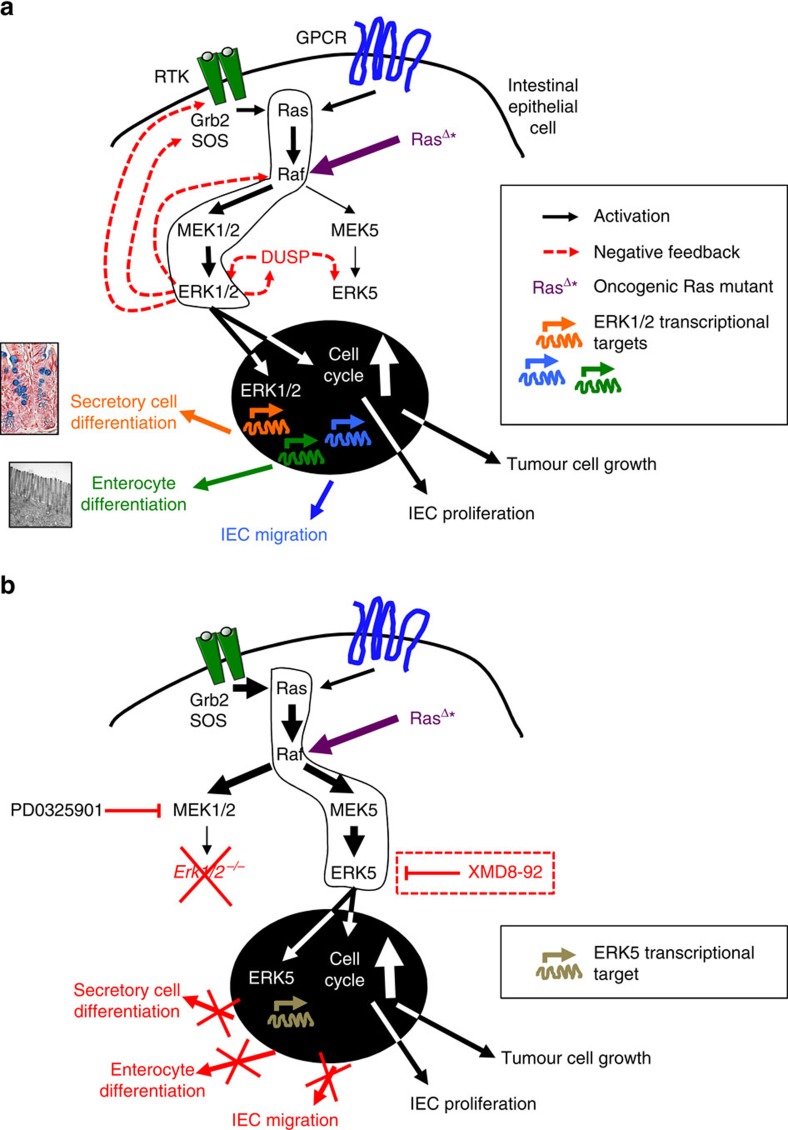
Roles of ERK1/2 and ERK5 in intestinal homeostasis and tumorigenesis. (**a**) When the ERK1/2 pathway is intact, extracellular cues that are transduced via RTKs or GPCRs activate Ras under physiological conditions, or alternatively, Ras is constitutively active in colorectal cancer (RasΔ*), which preferentially activates the Raf–MEK1/2–ERK1/2 module. The nuclear and transcriptional targets of ERK1/2 are crucial for enterocyte and secretory cell differentiation, IEC migration, as well as cell proliferation under homeostatic and oncogenic conditions. Importantly, ERK1/2 activation also results in the activation of negative feedback mechanisms that suppress its upstream kinases (for example, RTKs, son of sevenless, Raf) and activate dual specificity phosphatases (DUSPs), resulting in the silencing of the ERK5 module. (**b**) Upon abrogation of MEK1/2 or genetic knockout of *Erk1/2*, the lack of negative feedback mechanisms (that is, feedback activation) results in upregulation of the Ras–Raf–MEK5–ERK5 module, which maintains cell proliferation under physiological conditions, or results in continued tumour cell proliferation in colorectal cancer, respectively. However, the lack of activation of ERK1/2-specific targets results in differentiation and migration defects of intestinal epithelial cells culminating in malabsorption, wasting disease and mortality. Compensatory upregulation of the ERK5 pathway in CRC can be reversed by targeted treatment with its specific inhibitor, XMD8-92.

Migration and differentiation are functionally intertwined in the intestines, as demonstrated by the immature phenotype of mislocalized Paneth cells observed in ΔIEC mice (Fig. 2). Critical to epithelial cell migration is proper cytoskeleton reorganization mediated by the small GTPases of the Rho family, cell polarization regulated by Cdc42 and dynamic adhesion through cell–matrix and cell–cell interaction via integrin/FAK/Src signalling^31^. The ERK1/2 module is used as a downstream effector of many of these pathways in the intestine, including Rho GTPases^32^, FAK^33^ and Src^34^, and has been suggested to promote cell motility^33^,^35^. RTK signalling also contributes to cell migration, for example, Eph–Ephrin receptor interactions are crucial for correct positioning of Paneth cells^36^. Ephrin receptor-induced epithelial cell migration has been shown to be mediated by Src and ERK1/2 activation^37^,^38^, which may explain the Paneth cell mislocalization observed in ΔIEC mice. In summary, the ERK1/2 module is indispensable for full maturation of both absorptive enterocytes and the secretory lineage (Fig. 7a), confirming its crucial role in the integration of cellular cues required for determination of epithelial cell fate.

An unexpected finding was the redundancy of ERK1/2 in the gut with regard to cell proliferation. *Erk1/2* deletion was compensated by upregulated ERK5 signalling. Genetic targeting of ERK1/2 *in vitro* previously showed that *Erk2* knockdown is more effective than *Erk1* knockdown in suppressing cell proliferation, although this may be related to higher expression levels of the former^39^. The effect of gene dosage was demonstrated *in vivo* by the observations that while *Erk1*^*−/−*^ mice are viable^12^ and *Erk2*^*−/−*^ mice die *in utero*^13^, *Erk2*^*+/−*^ mice are only viable when at least one copy of *Erk1* is present. However, mice heterozygous (+/−) for both *Erk1* and *Erk2* alleles were born at lower than Mendelian ratio^39^. More recently, it was reported that transgenic expression of ERK1 can compensate for *Erk2* deletion^40^, demonstrating functional redundancy between both family members. Deletion of *Erk1/2* in adult skin tissue resulted in hypoplasia, which was associated with G2/M cell cycle arrest, without notable differentiation defects of keratinocytes^41^. These data differ from our observations in the intestines, which might be explained by incomplete and transient siRNA-mediated knockdown of ERK1/2 in primary keratinocyte cultures^41^, compared with more efficient genomic deletion of *Erk1* and *Erk2* that is typically achieved by the Villin-Cre-ERT2 system^14^, possibly resulting in different outcomes.

Both ERK1/2 and ERK5 have been described to promote cell cycle progression, although they have different upstream signalling partners, MEK1/2 and MEK5, respectively^1^. Furthermore, ERK2 and ERK5 proteins share only about 66% sequence identity, and MEK5 is phosphorylated by MEKK2/3, which can also activate the p38 and JNK pathways^42^. The ERK5 pathway is classically activated by stress stimuli, in addition to mitogens; thus, it shares features of both the ERK1/2, and p38 and JNK pathways, respectively^43^. ERK5 induces expression of cyclin D1 (refs 44, 45), and suppresses expression of cyclin dependent kinase inhibitors^46^, thereby promoting G1/S-phase cell cycle progression. Importantly, the role of ERK5 in IEC differentiation and intestinal homeostasis is currently unknown. Knockout of *Erk1/2* in IEC induced activity of ERK5, which was not detectable in naive mice (Fig. 3). These data suggest that the ERK1/2 and ERK5 modules may share proximal signalling components. Although EGFR is a likely candidate in this context^19^,^20^, we found that abrogation of EGFR signalling did not prevent enhanced ERK5 activity upon MEK1/2 inhibition. Although it was originally suggested that ERK5 signalling is independent of Ras^20^, other groups established that Ras, either through physiological signalling^47^, or by its oncogenic activity^48^,^49^, activates the MEK5–ERK5 signalling axis. Thus, rewiring of signalling networks downstream of Ras could explain the supraphysiological activity of ERK5 upon conditional deletion of *Erk1/2* in the intestines. In fact, it has been shown that ERK1/2 signalling mediates negative feedback on ERK5 activity^50^, possibly through transcriptional activation of dual specificity phosphatases (DUSPs)^51^. Alternatively, ERK1/2-induced FOS-like antigen 1 (Fra-1) may negatively regulate MEK5 (ref. 52). These data suggest that ERK5 is a default bypass route downstream of RTK-Ras and activated upon loss of ERK1/2-mediated repression, thereby ensuring the transduction of mitogenic signals to the nucleus (Fig. 7b). Consistent with this concept, we found that ERK5 inhibition induces atrophy of ΔIEC intestinal organoids (Fig. 4). In addition, important downstream transcriptional targets of ERK5 and ERK1/2 overlap, such as immediate-early gene *Fra1* and oncogene *c-Myc*, whereas *c-Fos* and *Egr1* were specifically induced by ERK1/2 (Fig. 6 and Supplementary Fig. 7). Specificity of ERK1/2 over ERK5 and other MAPK family members for the activation of c-Fos has been previously described^53^, demonstrating their differential biological output despite the shared ability to transduce potent mitogenic signals.

Our findings may be relevant for the use of MAPK inhibitors in the treatment of colorectal cancer. Although there was only a mild phenotype in the colons of ΔIEC mice under homeostatic conditions, the Ras–RAF–MEK–ERK pathway is generally upregulated in malignant cells including CRC^54^. Targeted therapy typically results in feedback activation of upstream players of the targeted kinase, which are then able to reactivate the same pathway or utilize bypass signalling routes^55^. For example, on activation, ERK1/2 phosphorylates EGFR, son of sevenless^56^, and Raf^57^, thereby terminating upstream signalling activity. Knockout of *Erk1/2* eliminates this negative feedback. Our data suggest that ERK5 is a putative resistance pathway in the context of targeted treatment with MEK1/2 or ERK1/2 inhibitors (Fig. 7b). Different classes of MEK1/2 inhibitors display various modes of resistance to therapy (innate, adaptive and acquired)^58^. Since we have only used one MEK1/2 inhibitor (PD0325901) in our studies, it will be necessary to evaluate other classes of inhibitors in combination with ERK5 inhibitors. Importantly, while treatment with either the MEK1/2 or ERK5 inhibitor suppressed tumour growth in murine *Apc*^*−/−*^ organoids, only the latter was able to inhibit the proliferation of *Apc*^*−/−*^*;KRAS*^*G12V*^ organoids (Fig. 6), which are more representative of human CRC. In line with this, suppression of ERK5 expression by forced expression of miR-143/145 inhibited intestinal adenoma formation in the *Apc*^*Min/+*^ model^59^, and activated MEK5 correlated with more invasive CRC in human^60^. ERK5 has been previously reported to mediate resistance to cytotoxic chemotherapy-induced apoptosis^61^. The highly specific and bioavailable ERK5 inhibitor, XMD8-92, has shown antitumour effects in multiple preclinical cancer models by inhibiting tumour angiogenesis, metastasis and chemo-resistance^62^. Furthermore, ERK5 inhibition does not induce feedback activation of upstream or parallel signalling pathways^62^. In conclusion, the ERK1/2 and ERK5 MAPK modules display a high degree of signalling plasticity in the intestinal epithelium, which has implications for targeted treatment of colorectal cancer.

## Methods

### Reagents

Tamoxifen, safflower oil, DL-dithiothreitol (DTT), DMSO and fluorescein isothiocyanate (FITC)-Dextran (FD-4) were purchased from Sigma. EDTA was obtained from Invitrogen. MEK1/2 inhibitor PD0325901 was obtained from Stemgent. ERK5 inhibitor XMD8-92 was obtained from Santa Cruz Biotechnology. EGFR inhibitor erlotinib was obtained from Selleckchem. Recombinant mWnt3a was obtained from R&D Systems.

### Antibodies

Anti-β-catenin (9562), anti-cyclin D1 (2922), anti-E-Cadherin (24E10), anti-phospho-ERK1/2 (T202/Y204; D13.14.4E), anti-ERK1/2 (137F5), anti-ERK5 (D23E9), anti-phospho-JNK (T183/Y185; 81E11), anti-JNK (9252), anti-MMP7 (3801), anti-phospho-p38 (T180/Y182; D3F9), anti-p38 (9212), anti-phospho-p90RSK (T359/S363; 9344), anti-p90RSK1/2/3 (9347), anti-TCF4 (C9B9) and anti-PCNA (PC10) antibodies were obtained from Cell Signaling Technologies. Anti-β-actin antibody (AC-74) and anti-α-Tubulin (T9026) came from Sigma, Alexa Fluor 488-conjugated claudin-1 (2H10D10) antibody from Invitrogen, anti-claudin-3 and anti-claudin-5 from Thermo Scientific, anti-phospho-ERK5 (T218/Y220; 07-507) antibody from Millipore, anti-Ki67 (GTX16667) antibody from GeneTex and anti-LYVE1 (ab33682) antibody from Abcam, anti-mucin 2 (H-300) antibody and anti-ERK2 (C-14) antibody from Santa Cruz Biotechnology. Antibodies for western blotting were used at 1:1,000 dilution. All uncropped western blots can be found in Supplementary Fig. 8.

### Cell culture and siRNA treatments

HCT116, DLD-1, Caco2, HT-29 and SW480 cell lines were obtained from ATCC and cultured in high-glucose DMEM (Mediatech), supplemented with 4 mM glutamine, 50 U ml^−1^ penicillin, 50 μg ml^−1^ streptomycin (all from Invitrogen) and 10% fetal calf serum. The cell lines were routinely tested for mycoplasma contamination with the MycoAlert Mycoplasma Detection Kit (Lonza). For cell line authentication, short tandem repeat (STR) analysis was performed on isolated genomic DNA with the GenePrint 10 System from Promega, and peaks were analysed using GeneMarker HID from Softgenetics. Allele calls were searched against STR databases maintained by ATCC (www.atcc.org), DSMZ (www.dsmz.de) and Texas Tech University Children's Oncology Group (cogcell.org). For some experiments, the cells were pre-treated with PD0325901, XMD8-92 or DMSO (control) before western blotting or cell viability assays. For siRNA-mediated knockdown, the cells were plated at 5 × 10^3^ cells per well in 96-well plates and transfected with 40 nM siRNA with optimized Dharmacon transfection reagents (Thermo). Oligonucleotides used: siGENOME non-targeting Control siRNA #5, siCONTROL TOX, ON-TARGETplus SMARTPOOL human CTNNB1 (all from Thermo), SignalSilence Erk1/2 (6560) and SignalSilence ERK5 siRNA I (7301) (Cell Signaling Technologies).

### Cell viability and reporter assays

XTT assays were performed according to the manufacturer's instructions (Biotium). In short, HCT116, DLD-1, HT-29 or SW480 cells were plated at 2–4 × 10^3^ cells per well (*n*=8 per condition) in 96-well plates (Corning) and XTT assays were performed on five consecutive days. For TOPflash luciferase reporter assays, HCT116 or SW480 cells were co-transfected with the TCF Reporter Plasmid (Millipore) and pcDNA3.1(+) vector (Thermo Scientific) that contains a Geneticin-selectable marker. Stable lines were selected with 800 μg ml^−1^ Geneticin (Thermo). The cells were lysed in total cell lysis buffer and Luciferase substrate (Promega) was added, followed by measurement of bioluminescence.

### Mice

Eight-to-12-week-old mice on the C57BL/6 background and bred in the animal facility of the Stein Clinical Research building at the University of California, San Diego were used for all the experiments, except for *Erk5*^*fl/fl*^ mice that were solely used for the generation of *ex vivo* intestinal organoids. Age-matched male and female mice were used for experiments. To generate the *Erk1*^*−/−*^*;Erk2*^*fl/fl*^;*Vil-Cre*^*ERT2*^ mouse strain, *Erk1*^*−/−*^;*Erk2*^*fl/fl*^ mice (a gift of Dr S. Hedrick, UCSD)^13^ were intercrossed with *Vil-Cre*^*ERT2*^mice that express the *CRE* transgene under control of the IEC-specific *Villin* promoter, which is inducible by tamoxifen^14^. Genotyping of the *Erk1* and *Erk2* genes, as well as the *CRE* transgene, with gDNA obtained from intestinal epithelial cells, skin and spleen tissue was used to confirm the genotype of the mice. *Erk1*^*−/−*^*;Erk2*^*fl/fl*^;*Vil-Cre*^*ERT2*^ mice were fertile and viable, and did not shown any overt phenotype without tamoxifen treatment. To induce recombination of the *Erk2* gene (deletion of exon 3) in IEC, the mice were treated with tamoxifen (1 mg day^−1^) dissolved in safflower oil for five consecutive days by oral gavage. The *Erk5*^*fl/fl*^ mice were generated by Dr C. Tournier (University of Manchester, Manchester, UK)^23^,^24^, and transferred from Dr Z. Xia's laboratory (University of Washington, Seattle, WA, USA). Gut barrier assays were performed with FITC-Dextran or by faecal albumin measurements with the Mouse Albumin ELISA kit (Bethyl Laboratories), as previously described^63^. All the experimental procedures were conducted in accordance with the University of California, San Diego institutional guidelines for Animal Care and Use (IACUC).

### Mouse blood analysis

Analysis of blood chemistry from blood plasma (albumin, globulin, total protein, liver enzymes, glucose, electrolytes, ALP, lipid spectrum, blood urea nitrogen) and haematological parameters (lymphocyte counts, haemoglobin) from EDTA blood were performed by the STAT Veterinary Lab, San Diego, CA, USA.

### IEC isolation

IEC isolation was performed as previously described^64^. Briefly, the intestines were washed three times in PBS with 1 mM dithiothreitol, followed by incubation in HBSS with 5 mM EDTA and 0.5 mM dithiothreitol and harvest of epithelial cells.

### RNA extraction and Q-PCR

For the preparation of RNA samples from murine IEC, the cell lysates were prepared with RLT buffer after IEC isolation procedures and stored at −80 °C until extraction. For RNA isolation from small intestinal organoid cultures, purified IEC were recovered from Matrigel using the BD Cell Recovery Solution following the manufacturer's instructions and cell lysates were prepared with RLT buffer. RNA extraction was performed with the RNeasy Mini Kit (Qiagen), followed by cDNA synthesis with the qScript cDNA superMix kit (Quanta Biosciences). Q-PCR was performed on the AB7300 Real-Time PCR System (Applied Biosystems) using PerfeCta SYBR Green FastMix (Quanta Biosciences). Oligonucleotide sequences for Q-PCR were custom designed with NCBI Primer-BLAST and synthesized by IDT Technologies (see Supplementary Table 1). For selected experiments, PCR products were run in TAE buffer on 2% agarose gels and visualized with SYBR Safe DNA (Invitrogen).

### Intestinal organoid culture

Small intestinal organoids were generated following published protocols^22^, and as previously described^64^. Briefly, intestinal organoids were grown in Advanced DMEM/F12 Reduced Serum Medium supplemented with 2 mM Glutamax, 10 mM HEPES, 100 U ml^−1^ penicillin and 100 μg ml^−1^ streptomycin, B27 and N2 supplements, 50 ng ml^−1^ mEGF (all from Invitrogen), 100 ng ml^−1^ mNoggin (PeproTech) and 10% (v/v) RspoI conditioned medium from 293 T-HA-RspoI-Fc cells (kindly provided by Dr C. Kuo, Stanford University). Growth Factor Reduced (GFR) Matrigel came from BD Biosciences. For experiments with *Erk5*^*−/−*^ intestinal organoids, small intestines were harvested from *Erk5*^*fl/fl*^ mice^23^,^24^ and stable organoid lines were generated. These were then transduced with Puro-Cre-ERT2 retrovirus (Addgene plasmid 22776; ref. 65) or Puro-Cre lentivirus (Addgene plasmid 17408) with a selectable marker (puromycin) following published protocols^66^. After transduction and puromycin selection, organoids were used for experiments with 4-hydroxytamoxifen (4-OHT) (Sigma) and/or PD0325901 treatment or western blotting. For experiments with *Apc*^*−/−*^ intestinal organoids, small intestines were harvested from *Apc*^*fl/fl*^ mice (kindly provided by Dr E. Fearon, University of Michigan Medical School)^25^, and stable organoid lines were generated. To delete the floxed *Apc* alleles, these organoids were infected with Adeno-Cre virus as previously described^67^. For experiments with *Apc*^*−/−*^;*KRAS*^*G12V*^ intestinal organoids, *Apc*^*−/−*^ intestinal organoids were transduced with *KRAS*^*G12V*^ lentivirus (Addgene plasmid 35633) with hygromycin. After transduction and selection, *Apc*^*−/−*^ and *Apc*^*−/−*^;*KRAS*^*G12V*^intestinal organoids were used for experiments with PD0325901 and/or XMD8-92 treatment.

### Immunohistochemistry and *in situ* hybridization

Immunohistochemistry was performed using standard staining procedures^64^. Briefly, 4–6-μm paraffin sections from murine small or large intestinal tissues were incubated with 1:200 dilutions of anti-Ki67 or anti-MMP7 antibodies (overnight, 4 °C), followed by detection with biotinylated secondary antibodies (1 h at room temperature; 1:500 dilution) and horseradish peroxidase-streptavidin conjugates (Jackson; 30 min at room temperature), followed by the addition of 3,3′-diaminobenzidine (DAB; Vector Laboratories). Counterstaining was performed with haematoxylin 560 (Surgipath). Alcian Blue (American MasterTech) and alkaline phosphatase (Vector Laboratories) stainings were performed according to the manufacturer's instructions. Oil red O stainings were performed by treating frozen sections with 60% isopropanol, followed by staining with Oil red O for 15 min, followed by rinsing with 60% isopropanol and haematoxylin counterstaining. For BrdU pulse-chase experiments, the mice were injected with BrdU (2 mg intraperitoneally) at 2, 24 or 48 h before analysis. The BrdU *In-Situ* Detection Kit (BD) was used for the detection of BrdU-labelled cells according to the manufacturer's instructions. Quantification of BrdU+ cells along the crypt–villus axis was performed following published methods^11^,^68^. *In situ* hybridization was performed to detect Cryptdin-1 expression to visualize Paneth cells in small intestinal sections, as previously described^69^.

### Immunofluorescent staining and confocal microscopy

Immunofluorescent staining and confocal imaging was performed using standard techniques^64^,^70^, by using Alexa Fluor (AF) 488 conjugated primary antibodies (1:200 dilution) with or without AF 546-conjugated phalloidin (1:500 dilution) and Hoechst 33258 (Invitrogen; 1:1,000 dilution) counterstaining. In some cases, AF 488-conjugated secondary antibodies (1:500 dilution) were used for visualization of the primary target (Molecular Probes). For immunofluorescent stainings of intestinal organoids, the cells were fixed in Matrigel, followed by incubation with 1:100 dilutions of primary antibodies and detection with AF 488-conjugated secondary antibody (1:500 dilution). Fluorescence images were acquired using × 20 air or × 63 oil-immersion objectives on a confocal laser-scanning microscope (Olympus IX81).

### TUNEL assay

Performed as previously described^11^ following the manufacturer's instructions (BD Biosciences).

### Flow cytometry

Flow cytometry analysis of intestinal epithelial cells was performed using standard protocols^11^,^70^. Propidium iodide (PI) stainings were performed with the FxCycle PI/RNase Staining Solution (Molecular Probes) following the manufacturer's instructions. Briefly, the cells were fixed with 4% paraformaldehyde and prepared at 1 × 10^6^ cells per condition. The cells were pelleted, followed by the addition of 0.5 ml of PI/RNase Staining Solution. After a 15-min incubation period, the samples were run on a BD Accuri C6 flow cytometer.

### Transmission electron microscopy

Transmission electron microscopy (TEM) was performed by using a Zeiss EM10C electron microscope with a Gatan 785 11 megapixel digital camera at the Microimaging Core Facility (VA San Diego Healthcare System). Mouse small intestinal samples were fixed with 2% paraformaldehyde with 2.5% glutaraldehyde, embedded in epoxy 812 and sectioned with a Reichert Ultracut ultramicrotome for placement on TEM grids.

### Statistical analysis

The data are represented as mean±s.e.m. or as stated in the figure legends. The *P* values are stated in the figure legends; a *P* value <0.05 was considered significant. Unpaired Student's *t*-test was used for statistical analyses to compare two data sets with normal distribution, Mann–Whitney *U*-test was used for nonparametric data, analysis of variance was used to compare multiple data sets, log-rank analysis was applied for survival curves. All the statistical tests were performed with Prism 6.0 (GraphPad, La Jolla, CA, USA).

## Additional information

**How to cite this article:** de Jong, P. R. *et al*. ERK5 signalling rescues intestinal epithelial turnover and tumour cell proliferation upon ERK1/2 abrogation. *Nat. Commun.* 7:11551 doi: 10.1038/ncomms11551 (2016).

## Supplementary Material

Supplementary Information Supplementary Figures 1-8 and Supplementary Table 1
