# Supplementary material for: ERK5 signalling rescues intestinal epithelial turnover and tumour cell proliferation upon ERK1/2 abrogation
Source: Nat Commun. 2016 May 17;7:11551. doi: 10.1038/ncomms11551 (PMC4873670; doi:10.1038/ncomms11551)
Supplement: Supplementary Information — Supplementary Figures 1-8 and Supplementary Table 1 [file ncomms11551-s1.pdf]

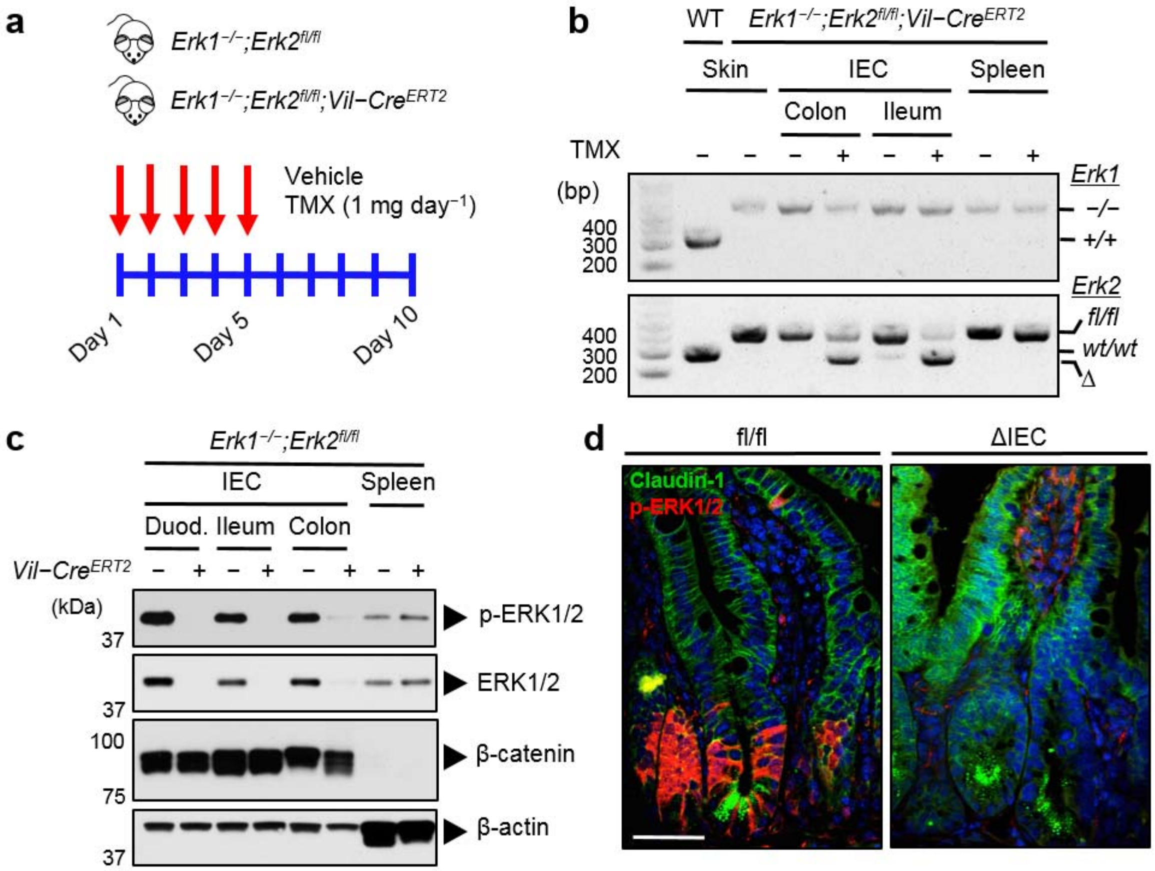

Supplementary Fig. 1. Validation of genetically engineered *Erk1*$^{-/-}$*;Erk2*$^{fl/fl}$*;Vil-Cre*^{ERT2} mice.

a, *Erk1*$^{-/-}$*;Erk2*$^{fl/fl}$ ("fl/fl") or *Erk1*$^{-/-}$;*Erk2*$^{fl/fl}$*;Vil-Cre*^{ERT2} ("ΔIEC") mice received 5 consecutive treatments with vehicle or tamoxifen (1 mg day^{-1}) by oral gavage, and were killed at day 10. **b**, Genotyping of *Erk1* and *Erk2* in various tissues from WT or *Erk1*$^{-/-}$;*Erk2*$^{fl/fl}$*;Vil-Cre*^{ERT2} mice treated with vehicle or tamoxifen. **c**, All the *Erk1*$^{-/-}$*;Erk2*$^{fl/fl}$ mice with or without transgene (*Vil-Cre*^{ERT2}) were treated as in **a**. Immunoblotting for indicated proteins with lysates of IECs (isolated from duodenum, ileum or colon) or splenocytes. β-catenin, an IEC marker. β-actin, loading control. **d**, Immunofluorescent staining for p-ERK1/2 and Claudin-1, an IEC marker, with small intestinal sections prepared from fl/fl and ΔIEC mice. Scale bar = 100 μm.

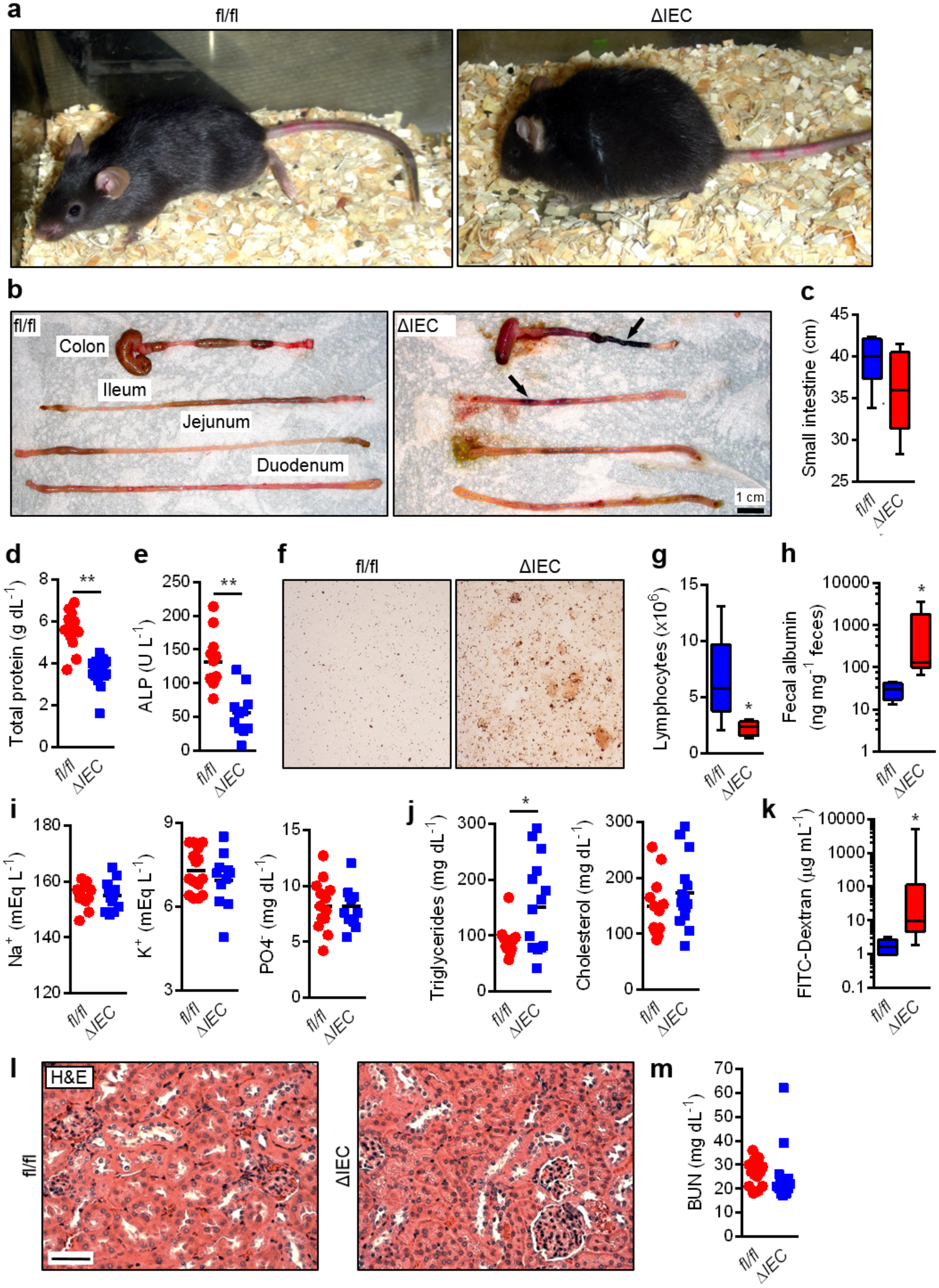

a
fl/fl
ΔIEC
b
fl/fl
Colon
Ileum
Jejunum
Duodenum
ΔIEC
1 cm
c
Small intestine (cm)
fl/fl
ΔIEC
d
Total protein (g dL-1)
**
e
ALP (U L-1)
**
f
fl/fl
ΔIEC
g
Lymphocytes (x106)
*
h
Fecal albumin (ng mg-1 feces)
*
i
Na+ (mEq L-1)
K+ (mEq L-1)
PO4- (mg dL-1)
j
Triglycerides (mg dL-1)
*
Cholesterol (mg dL-1)
k
FITC-Dextran (μg mL-1)
*
l
H&E
fl/fl
ΔIEC
m
BUN (mg dL-1)

Supplementary Fig. 2. Intestinal and metabolic consequences in *Erk1/2*$^{\Delta IEC}$ mice.

Comparisons between fl/fl and ΔIEC mice littermates performed at day 10 of follow-up after tamoxifen treatment. **a**, Moribund phenotype of ΔIEC mice compared with fl/fl littermates. Representative examples are shown. **b**, Macroscopic appearance of the small intestine and colon. Sites of macroscopic bleeding in the ileum and colon are indicated by arrows. **c**, Length of the small intestine of fl/fl (n=6) and ΔIEC (n=5) mice. **d, e**, Serum was analyzed for: total protein levels (**d**), and alkaline phosphatase (ALP) levels (**e**). **f**, Fecal smears stained with Oil red O (representative examples from each group). **g**, Lymphocyte counts in peripheral blood samples of fl/fl (n=12) and ΔIEC (n=10) mice. **h**, Albumin levels measured in stool samples of fl/fl (n=4) and ΔIEC (n=6) mice. **i**, Electrolyte levels measured in serum. **j,** Triglyceride and cholesterol levels measured in serum. **k**, Gut barrier assay by oral administration of FITC-Dextran (FD-4) and measurement of FD-4 in peripheral blood of fl/fl (n=7) and ΔIEC (n=11) mice. **l**, Histological features of the renal parenchyma in fl/fl and ΔIEC mice. Representative results are shown. Scale bar = 100 µm. **m**, Blood urea nitrogen (BUN) levels in fl/fl and ΔIEC mice. Data are represented as box-and-whiskers plots (**c, g-h, k**), or scatter plots with mean (**d-e, i-j, m**). $^*P<0.05$ and $^{**}P<0.001$ by Student's t test.

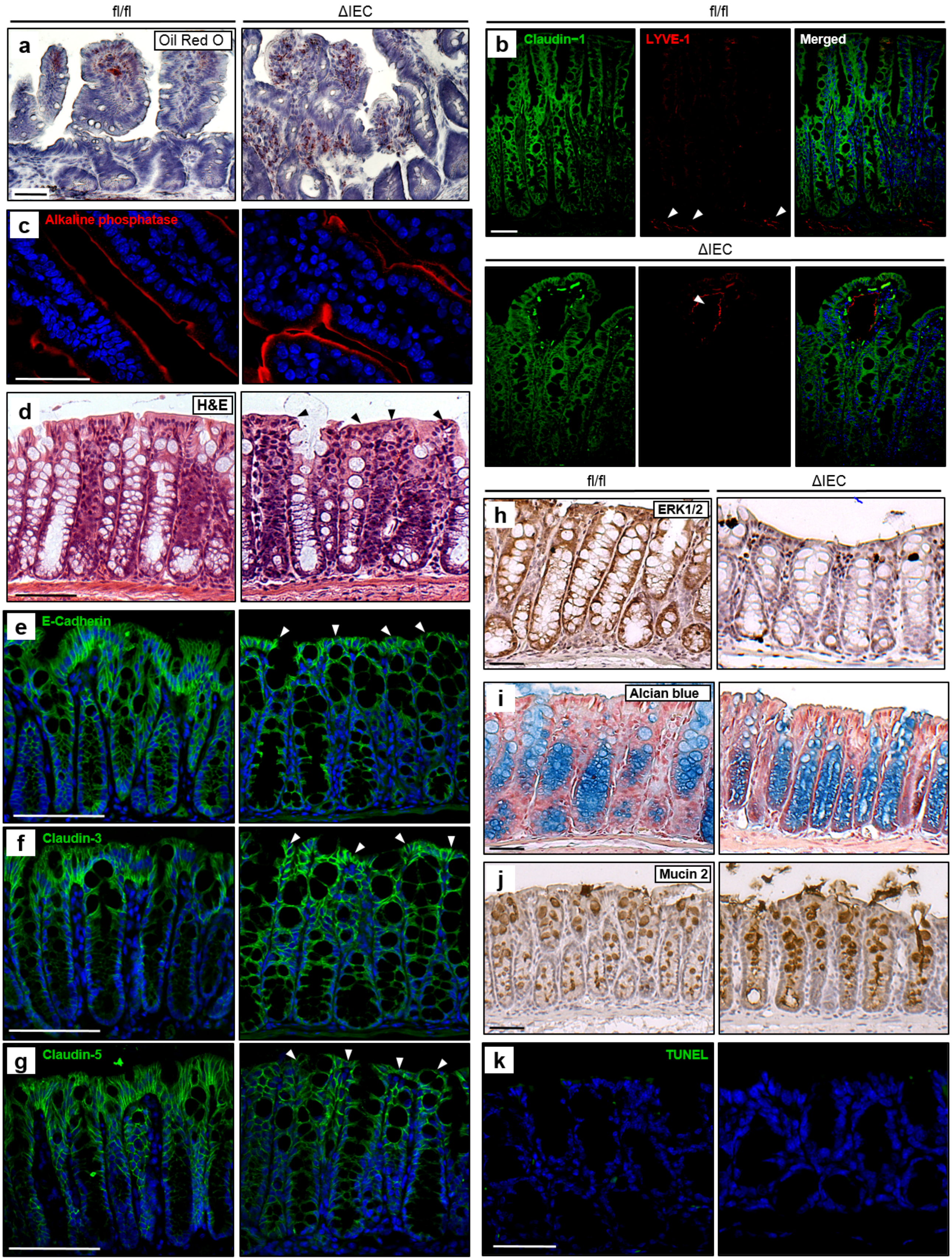

fl/fl
ΔIEC
fl/fl
a
Oil Red O
b
Claudin-1
LYVE-1
Merged
ΔIEC
c
Alkaline phosphatase
d
H&E
fl/fl
ΔIEC
h
ERK1/2
e
E-Cadherin
i
Alcian blue
f
Claudin-3
j
Mucin 2
g
Claudin-5
k
TUNEL

Supplementary Fig. 3. Intestinal abnormalities in *Erk1/2*$^{\Delta IEC}$ mice.

a-k, Comparisons of small intestine (ileum) and colon sections from fl/fl and ΔIEC mice 10 days after tamoxifen treatment. (**a**) Lipid staining with Oil red O of frozen sections from small intestine prepared from fl/fl and ΔIEC mice. Counter staining with hematoxylin. (**b**) Immunofluorescent staining of paraffin-embedded sections from the small intestine for a lymphatic endothelial cell marker, lymphatic vessel endothelial hyaluronan receptor lymph marker (LYVE-1), and an IEC marker, Claudin-1. (**c**) Alkaline phosphatase staining of ileum sections from fl/fl and ΔIEC mice. (**d**) Gross crypt architecture in colon sections prepared from fl/fl and ΔIEC mice (H&E staining). Immunofluorescent staining for adherens protein E-Cadherin (**e**), and tight junction proteins Claudin-3 (**f**) or Claudin-5 (**g**). Arrowheads indicate the loss of the flat, epithelial sheet in colons from ΔIEC mice which is normally visible in fl/fl (control) mice. Immunohistochemical staining for ERK1/2 (**h**), Alcian blue (**i**) or Mucin 2 (**j**) of colon sections from fl/fl and ΔIEC mice. (**k**) TUNEL assay performed with colon sections from fl/fl and ΔIEC mice. Scale bars = 50 (**k**) or 100 µm (**a-j**). Representative examples are shown.

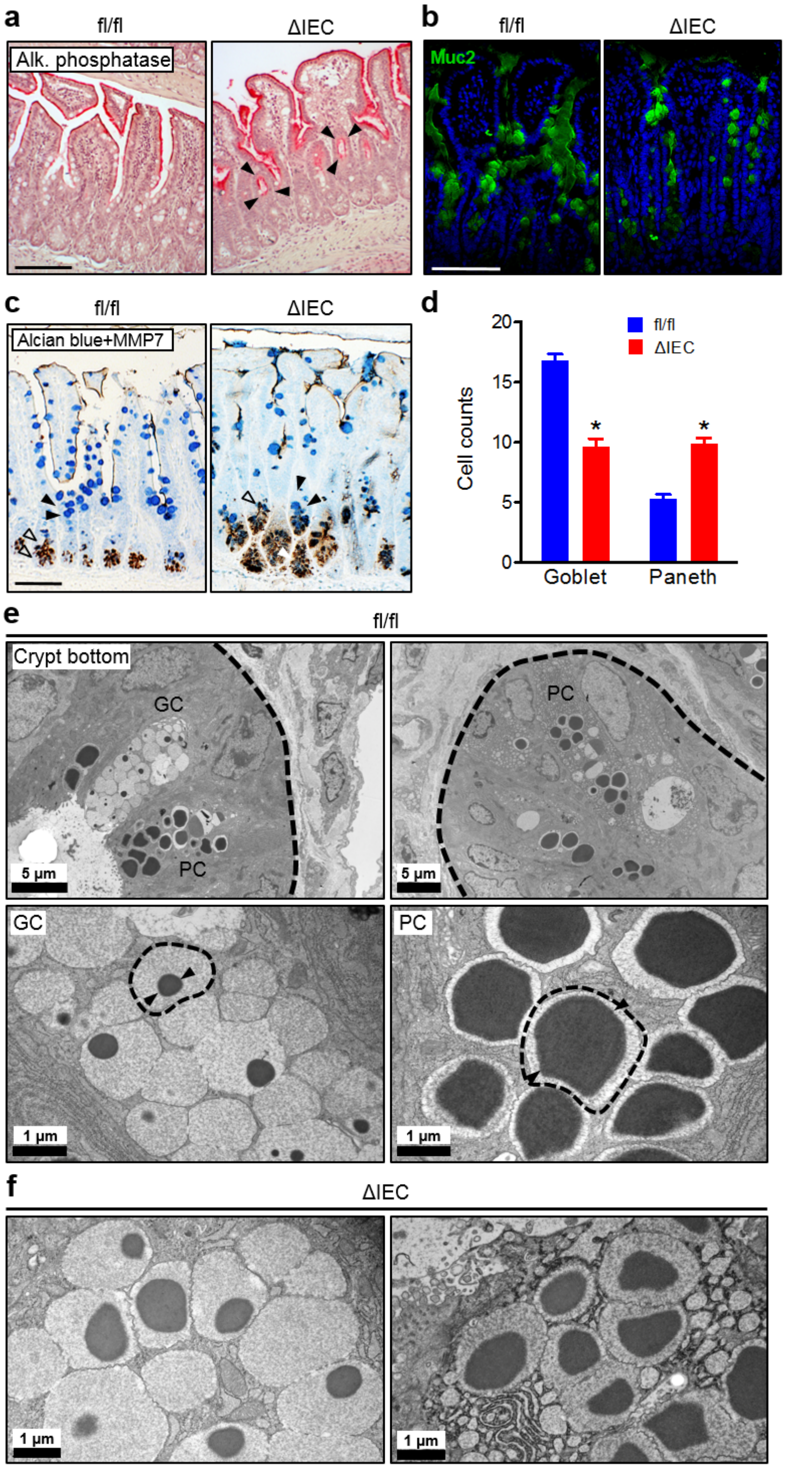

Supplementary Fig. 4. Abnormal secretory cell differentiation in *Erk1/2*$^{\Delta IEC}$ mice.

Comparisons between fl/fl and ΔIEC mice littermates performed at day 10 of follow-up after tamoxifen treatment. **a**, Alkaline phosphatase staining of paraffin-embedded ileum sections. Representative results are shown. **b**, Immunofluorescent staining of paraffin-embedded ileum sections for Muc2. Analysis by

fluorescence microscopy. **c**, Immunohistochemical staining of ileum sections with Alcian blue and anti-MMP7 antibodies, detected with DAB (brown). Open arrowheads indicate MMP7 positive cells at the crypt base; closed arrowheads indicate the most proximal Alcian blue positive staining cells along the crypt-villus axis. **d**, Enumeration of goblet and Paneth cells (per crypt-villus unit) by semi-quantitative, blinded scoring in ileum sections from fl/fl and ΔIEC mice (n=3 for each condition). Data are mean ± s.e.m. *P<0.05 by Student's t test. **e**, Top panels: visualization of cells residing in the crypt bottom in fl/fl mice by using transmission electron microscopy (TEM). Crypt outlines are indicated by dashed lines in top two panels (4000× and 3150× magnification, respectively). Bottom panels: high magnification images of a typical goblet (GC) and Paneth cell (PC) found in fl/fl mice. Dashed lines indicate granules, arrowheads indicate typical electrodense cores observed in mature cells (20000× magnification, TEM). **f**, Representative examples of the abnormal features of goblet cells and Paneth cells, respectively, typically found in ΔIEC mice. Scale bars = 100 μm (**a**-**c**), or as indicated (**e**-**f**).

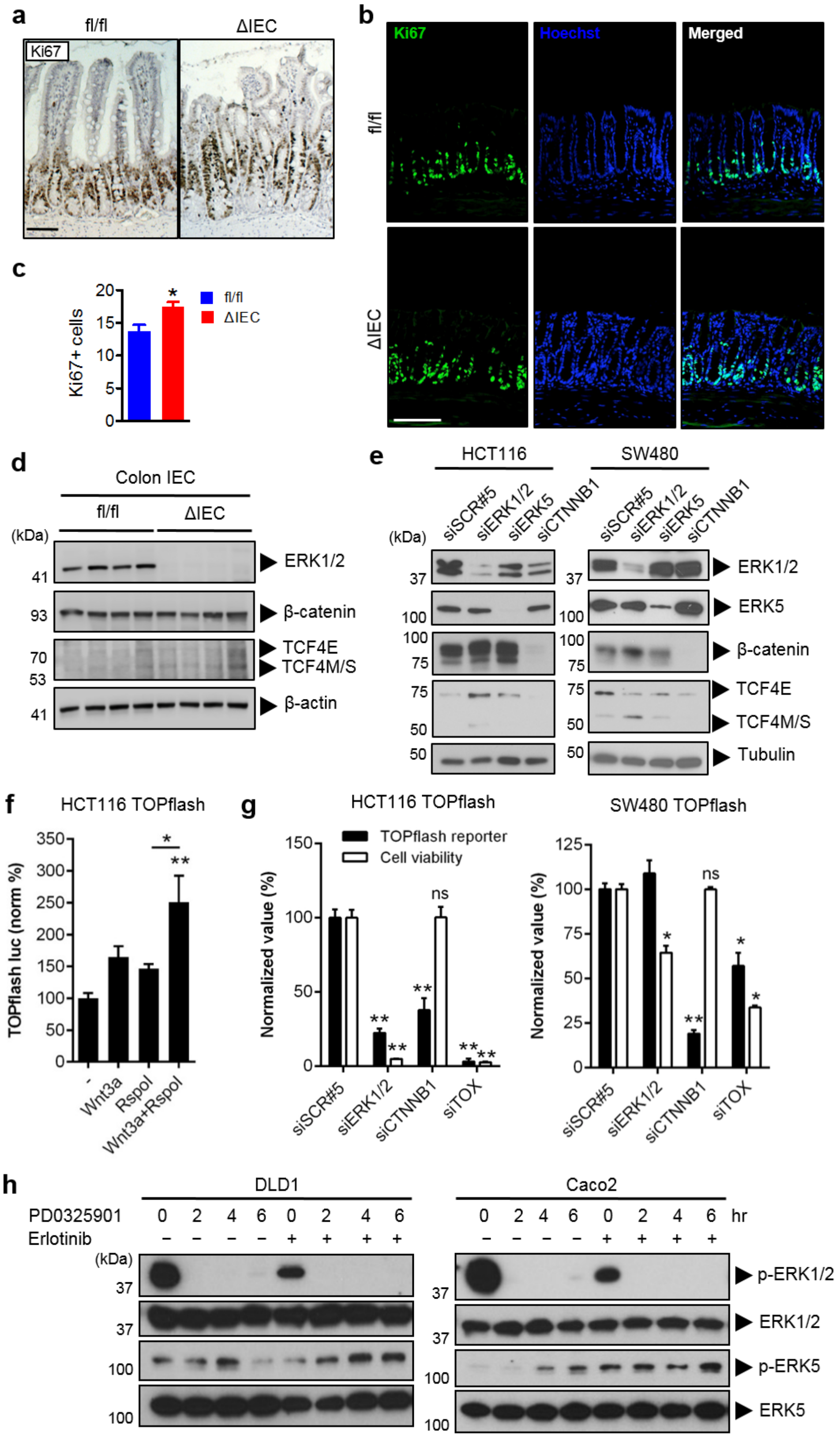
a
fl/fl
ΔIEC
Ki67
b
Ki67
Hoechst
Merged
fl/fl
ΔIEC
c
Ki67+ cells
fl/fl
ΔIEC
d
Colon IEC
fl/fl
ΔIEC
(kDa)
ERK1/2
β-catenin
TCF4E
TCF4M/S
β-actin
e
HCT116
SW480
siSCR#5
siERK1/2
siERK5
siCTNNB1
(kDa)
ERK1/2
ERK5
β-catenin
TCF4E
TCF4M/S
Tubulin
f
HCT116 TOPflash
TOPflash luc (norm %)
-
Wnt3a
Rspol
Wnt3a+Rspol
g
HCT116 TOPflash
SW480 TOPflash
TOPflash reporter
Cell viability
Normalized value (%)
ns
siSCR#5
siERK1/2
siCTNNB1
siTOX
h
DLD1
Caco2
PD0325901
Erlotinib
hr
(kDa)
p-ERK1/2
ERK1/2
p-ERK5
ERK5

Supplementary Fig. 5. *Erk1/2$^{\Delta IEC}$* mice display a hyperproliferative phenotype.

a, Immunohistochemical staining with paraffin-embedded ileum sections from fl/fl and ΔIEC mice, 10 days after tamoxifen treatment, for Ki67. Counter staining with hematoxylin. **b**, Immunofluorescent staining with paraffin-embedded colon sections from fl/fl and ΔIEC mice, as in **a**, for Ki67. **c**, Enumeration of the number of Ki67 positive cells (per crypt) in the colons of fl/fl and ΔIEC mice, respectively (n=3/group). Data are mean ± s.e.m. *P<0.05 by Student's t test. **d**, Colon IEC were isolated from tamoxifen-treated fl/fl and ΔIEC mice at day 10 of follow-up. Total cell lysates were analyzed by Western blotting for ERK1/2, β-catenin, TCF4 and β-actin. Representative results of three independent experiments. **e**, CRC cell lines HCT116 and SW480 were treated with siSCR#5 (non-targeting Control siRNA #5), siERK1/2, siERK5 or siCTNNB1 (40 nM) for 72 hr. Whole cell lysates were analyzed by Western blotting for abundance of ERK1/2, ERK5, β-catenin, TCF4 and Tubulin. **f**, SW480 and HCT116 cell lines were co-transfected with TOPflash and pcDNA3.1(+) plasmids and selected with Geneticin. To validate the specificity of reporter activity, HCT116 TOPflash cells were treated with Wnt3a ligand (100 ng/mL) and/or R-spondin 1 (RspoI) conditioned media (20% v/v) for 24 hr, followed by cell viability and luciferase assays. Data are mean ± s.e.m. *P<0.05 or **P<0.01 vs. control, or as indicated, by ANOVA. **g**, HCT116 and SW480 TOPflash reporter lines were treated with 40 nM siSCR#5 (non-targeting Control siRNA #5), siERK1/2, siCTNNB1 or siCONTROL TOX for 72 hr, after which luciferase activity and cell viability (XTT assay) were measured. Data are mean ± s.e.m. *P<0.05 or **P<0.01 vs. siSCR#5, by ANOVA. **h**, DLD1 and Caco2 cells were treated with the specific MEK1/2 inhibitor, PD0325901 (100 nM), for 0, 2, 4 or 6 hr with or without the EGFR inhibitor, erlotinib (10 μM). Cell lysates were analyzed by immunoblotting for indicated proteins. Scale bars = 100 μm (**a**, **b**).

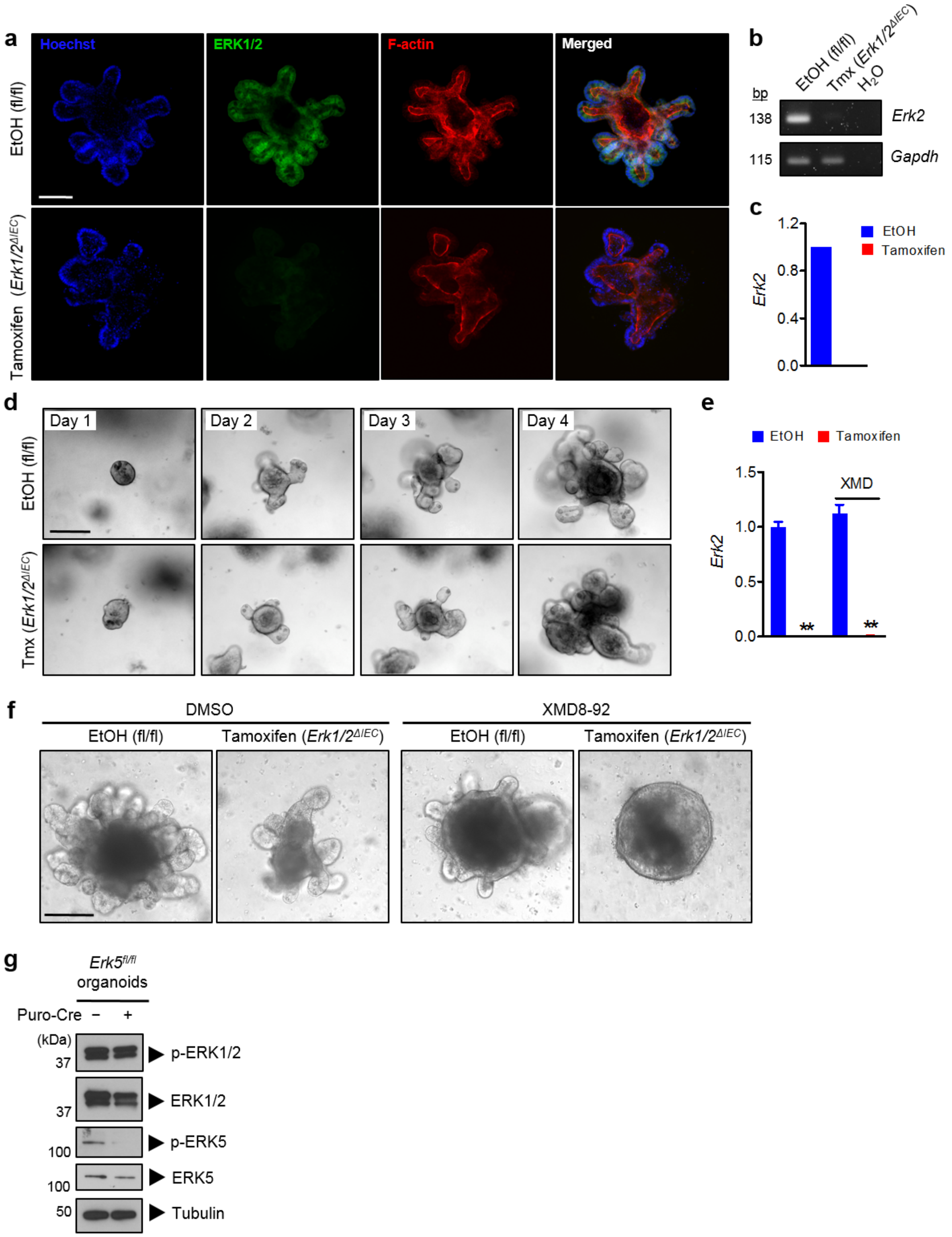
a
Hoechst
ERK1/2
F-actin
Merged
EtOH (fl/fl)
Tamoxifen (Erk1/2ΔIEC)
b
bp
EtOH (fl/fl)
Tmx (Erk1/2ΔIEC)
H2O
138
Erk2
115
Gapdh
c
1.2
0.8
0.4
0.0
Erk2
EtOH
Tamoxifen
d
Day 1
Day 2
Day 3
Day 4
EtOH (fl/fl)
Tmx (Erk1/2ΔIEC)
e
EtOH
Tamoxifen
1.5
1.0
0.5
0.0
Erk2
XMD
**
**
f
DMSO
XMD8-92
EtOH (fl/fl)
Tamoxifen (Erk1/2ΔIEC)
EtOH (fl/fl)
Tamoxifen (Erk1/2ΔIEC)
g
Erk5fl/fl
organoids
Puro-Cre
−
+
(kDa)
37
p-ERK1/2
37
ERK1/2
100
p-ERK5
100
ERK5
50
Tubulin

Supplementary Fig. 6. Genetic deletion of *Erk1/2* or *Erk5* in intestinal organoids.

a, Full images of intestinal organoids generated from ΔIEC mice and treated with EtOH or tamoxifen (0.5 μM), as shown in **Fig. 4a**. **b**, **c**, Analysis of *Erk2* transcript levels in ΔIEC organoids after EtOH or tamoxifen (0.5 μM) treatment. Q-PCR products were visualized by gel electrophoresis together with *Gapdh* (**b**). Quantification of *Erk2* expression in fl/fl and ΔIEC organoids by Q-PCR, normalized for *Gapdh* (**c**). **d**, Budding, growth and morphological characteristics of fl/fl and ΔIEC organoids after *ex vivo* treatment with EtOH or tamoxifen (0.5 μM) on day 1 and 2. **e**, **f**, ΔIEC organoids were treated with EtOH or tamoxifen (0.5 μM), with or without co-treatment with the ERK5 inhibitor, XMD8-92 (10 μM), respectively. Treatment was started 2 days after organoid passing for 2 consecutive days. Organoids were analyzed on day 5 of culture. Analysis of *Erk2* transcript expression by Q-PCR (n=2-3/group) with organoid lysates, normalized for *Gapdh* (**e**). Data are mean ± s.e.m. ***P*<0.01 versus control (EtOH) or as indicated, by ANOVA. Intestinal organoids with or without co-treatment were imaged by live-cell brightfield microscopy (**f**). **g,** Intestinal organoids generated from $Erk5^{fl/fl}$ mice were transduced with Puro-Cre lentivirus (empty vector). Transduced organoids were then selected with puromycin for 7 days. Whole cell lysates were prepared and analyzed by Western blotting for p-ERK1/2, ERK1/2, p-ERK5, ERK5 and Tubulin. Representative results of two independent experiments. Scale bars = 100 μm (**a**, **d**, **f**).

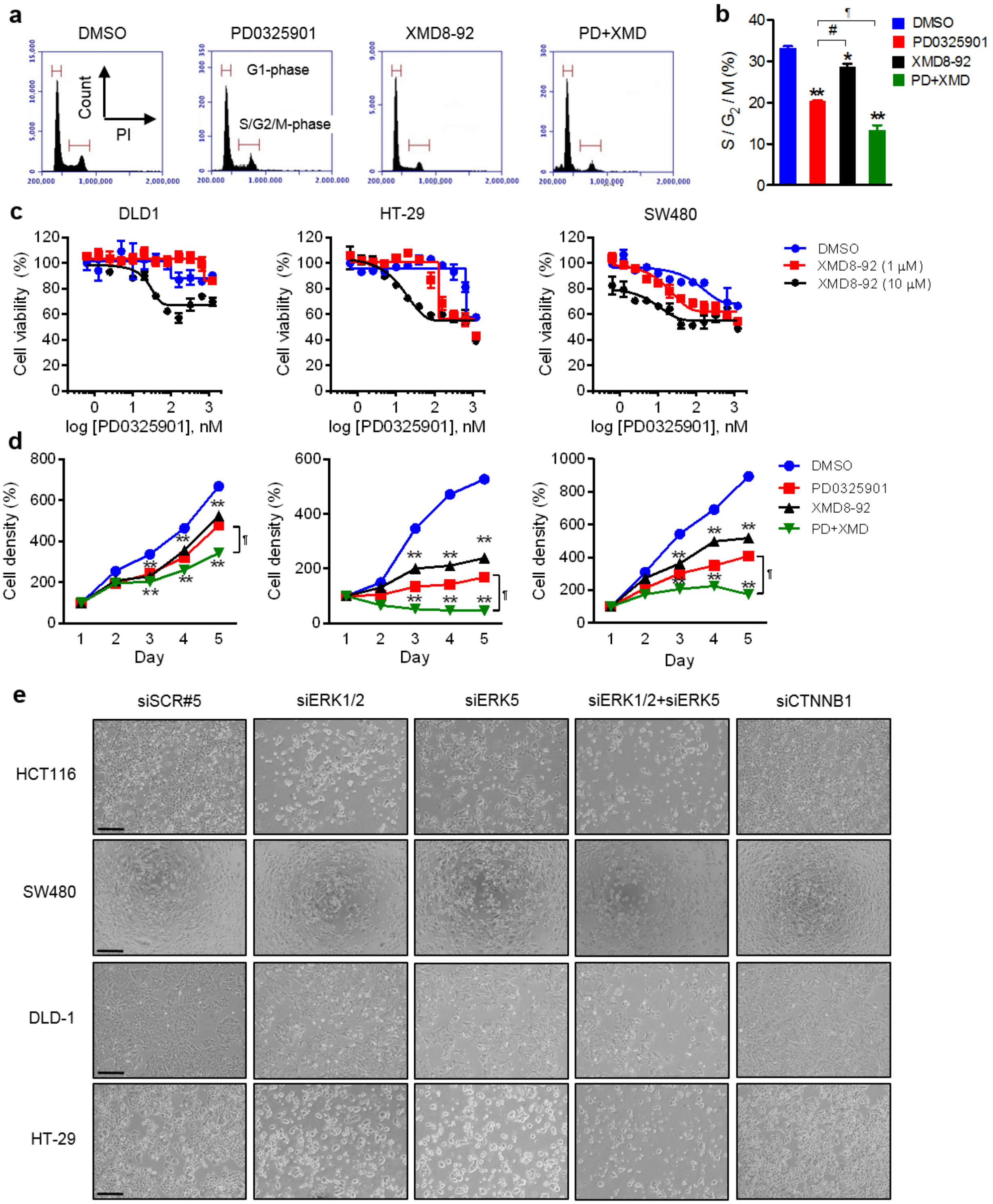
a
DMSO
PD0325901
XMD8-92
PD+XMD
Count
PI
G1-phase
S/G2/M-phase
b
S / G2 / M (%)
DMSO
PD0325901
XMD8-92
PD+XMD
c
DLD1
HT-29
SW480
Cell viability (%)
log [PD0325901], nM
DMSO
XMD8-92 (1 μM)
XMD8-92 (10 μM)
d
Cell density (%)
Day
DMSO
PD0325901
XMD8-92
PD+XMD
e
siSCR#5
siERK1/2
siERK5
siERK1/2+siERK5
siCTNNB1
HCT116
SW480
DLD-1
HT-29

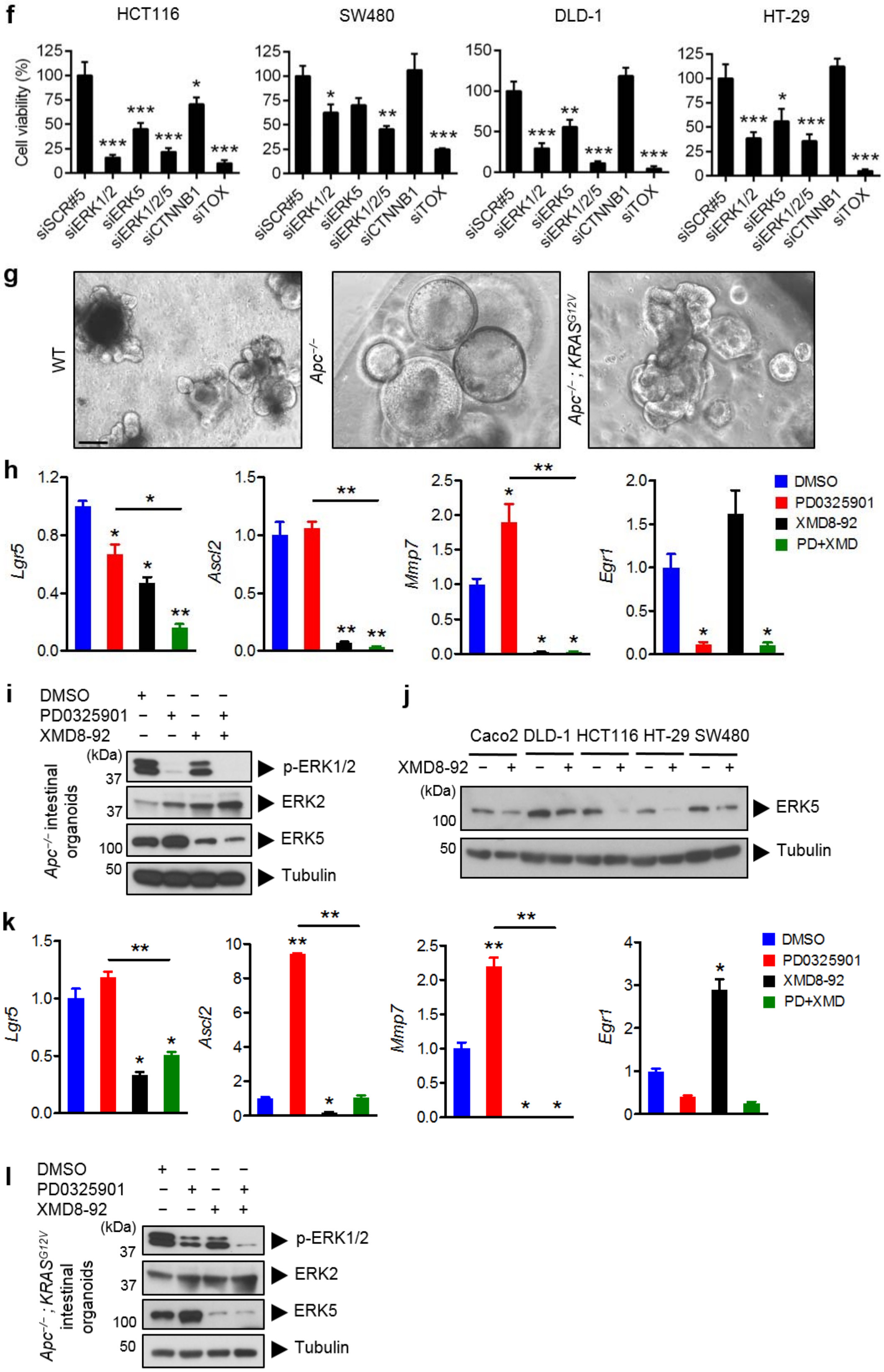

f
HCT116
SW480
DLD-1
HT-29
Cell viability (%)
siSCR#5
siERK1/2
siERK5
siERK1/2/5
siCTNNB1
siTOX
g
WT
Apc−/−
Apc−/−; KRASG12V
h
Lgr5
Ascl2
Mmp7
Egr1
DMSO
PD0325901
XMD8-92
PD+XMD
i
DMSO
PD0325901
XMD8-92
(kDa)
Apc−/− intestinal organoids
p-ERK1/2
ERK2
ERK5
Tubulin
j
Caco2 DLD-1 HCT116 HT-29 SW480
XMD8-92
ERK5
Tubulin
k
Lgr5
Ascl2
Mmp7
Egr1
DMSO
PD0325901
XMD8-92
PD+XMD
l
DMSO
PD0325901
XMD8-92
(kDa)
Apc−/−; KRASG12V intestinal organoids
p-ERK1/2
ERK2
ERK5
Tubulin

Supplementary Fig. 7. Dual inhibition of ERK1/2 and ERK5 inhibits intestinal tumor cell proliferation.

a, **b**, HCT116 cells were treated with DMSO, PD0325901 (100 nM), XMD8-92 (10 µM) or PD0325901 (100 nM) + XMD8-92 (10 µM), stained with propidium iodide 24 hr later and analyzed by flow cytometry. Representative examples of propidium iodide staining of HCT116 cells after treatment with pharmacological inhibitors (**a**). Quantification of cells in S-phase, G2 or M-phase in each experimental condition (n=3/condition) (**b**). **c**, DLD1, HT-29 and SW480 cells were plated at 5×10^3 cells/well and treated with PD0325901 (0.63, 1.25, 2.5, 5, 10, 20, 40, 80, 160, 320, 640 or 1280 nM) with or without XMD8-92 (0, 1, or 10 µM). Cell viability was assessed by XTT assay after 48 hr of treatment. **d**, DLD1, HT-29 and SW480 cells were plated at 2×10^3 cells/well and cultured in the presence of DMSO, PD0325901 (100 nM), XMD8-92 (10 µM) or PD0325901 (100 nM) + XMD8-92 (10 µM). Compounds were added on day 0 and day 2. XTT assay was performed on five consecutive days. Results are normalized to cell densities on day 1 (n=8/condition). **e**, **f**, CRC cell lines HCT116, SW480, DLD-1 and HT-29 were treated with 40 nM siSCR#5, siERK1/2, siERK5, siERK1/2+siERK5, siCTNNB1 or siTOX. Cell viability was measured after 72 hr by XTT assay. Representative light cell microscopy pictures of corresponding cell cultures Scale bars = 200 µm (**e**). Quantification of cell viability by XTT assay (**f**). **g**, Left two panels: Morphological characteristics of small intestinal organoids generated from wild-type mice, or $Apc^{fl/fl}$ mice and transduced *ex vivo* with Adeno-Cre ($Apc^{-/-}$), respectively. Right panel: Morphological features of $Apc^{-/-}$ organoids that were transduced with $KRAS^{G12V}$ expressing lentivirus *ex vivo*. Organoids were visualized by live-cell brightfield microscopy on day 5 after passage. Scale bar = 100 µm. **h**, **i**, Intestinal organoids generated from $Apc^{-/-}$ mice were treated with DMSO, PD0325901 (20 nM), XMD8-92 (10 µM) or PD0325901 (20 nM) + XMD8-92 (10 µM) for 5 days. Organoid cell lysates were analyzed by Q-PCR (n=3/condition) for intestinal stem cell markers, *Lgr5* and *Ascl2,* the Paneth cell marker, *Mmp7*, and the immediate-early gene, *Egr1* (**h**). Protein samples were analyzed by immunoblotting for indicated proteins (**i**). **j**, Immunoblotting of protein samples isolated from CRC lines Caco2, DLD-1, HCT116, HT-29 and SW480 after treatment with DMSO or XMD8-92 (10 µM) for 3 days. **k**, **l**, Intestinal organoids generated from $Apc^{-/-}$ mice were transduced with $KRAS^{G12V}$ expressing lentivirus, and treated with DMSO, PD0325901 (20 nM), XMD8-92 (10 µM) or PD0325901 (20 nM) + XMD8-92 (10 µM) for 5 days. Organoid cell lysates were analyzed by Q-PCR (n=3/condition) as in **h** (**k**), protein samples were analyzed by immunoblotting for indicated proteins (**l**). Data are mean ± s.e.m. *$P<0.05$, **$P<0.001$ versus DMSO or as indicated (**b**, **h, k**), # $P<0.01$ for XMD8-92 versus PD0325901 (**b**), ¶ $P<0.01$ for PD0325901 versus PD+XMD (**b**, **d**), *$P<0.05$, **$P<0.01$ or ***$P<0.001$ vs. siSCR#5 (**f**), by ANOVA.

Supplementary Fig. 8. Full original immunoblots and gel images.

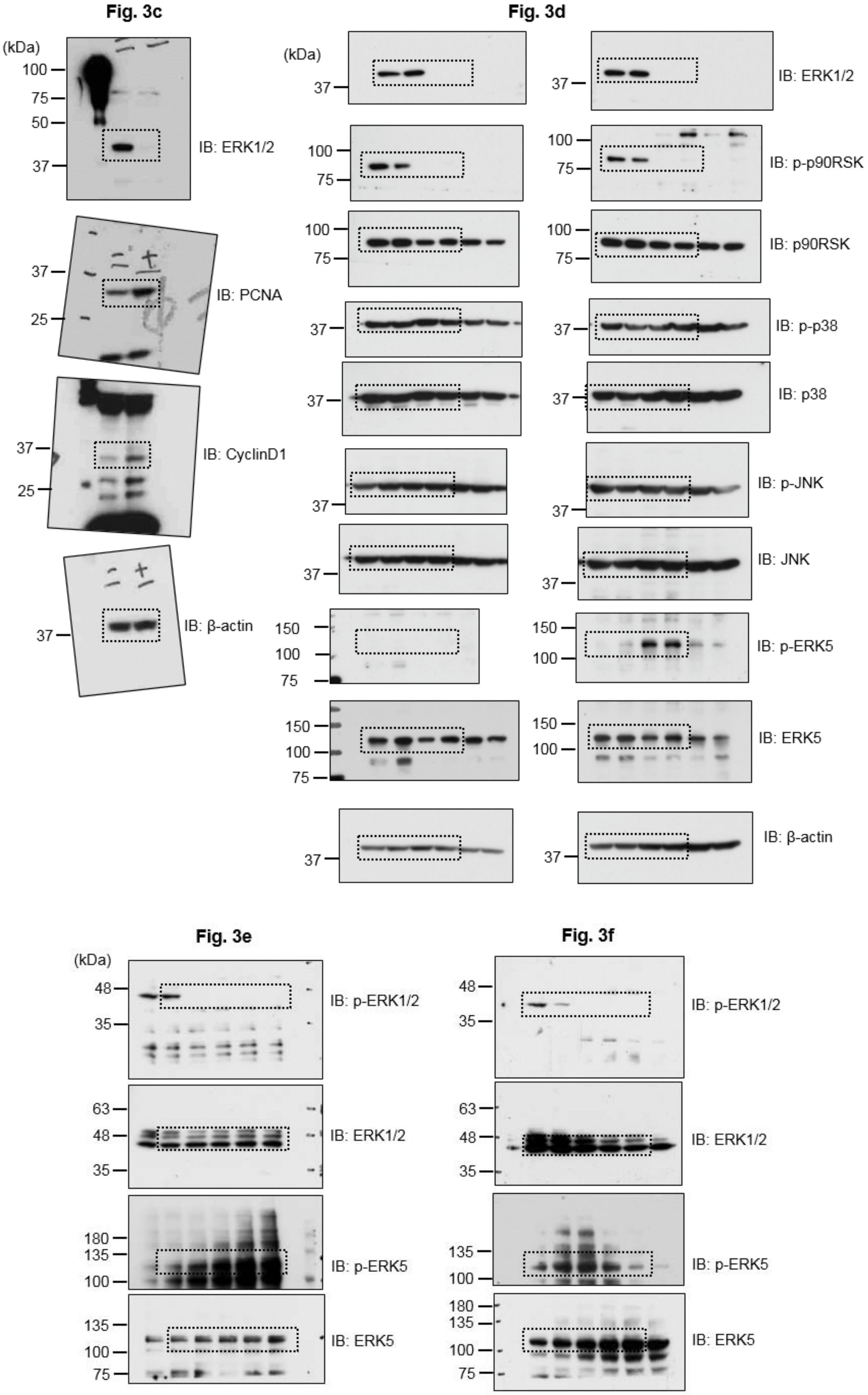

Fig. 3g

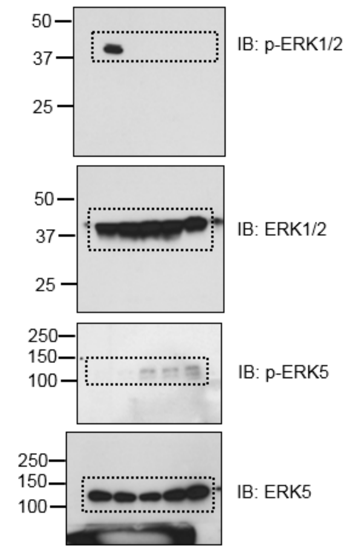

Fig. 3h

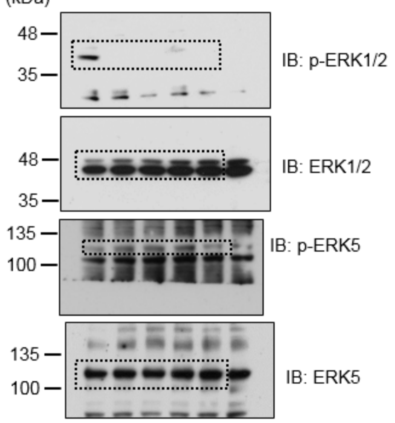

Fig. 3i

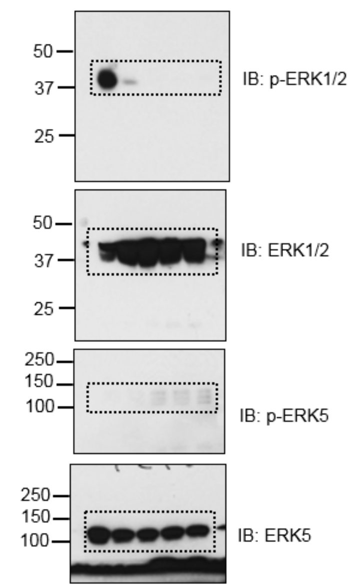

Fig. 3j

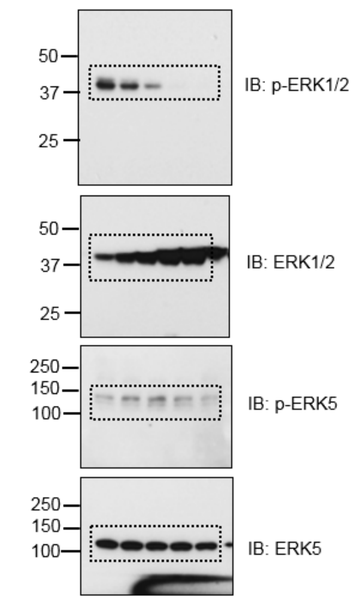

Fig. 4b

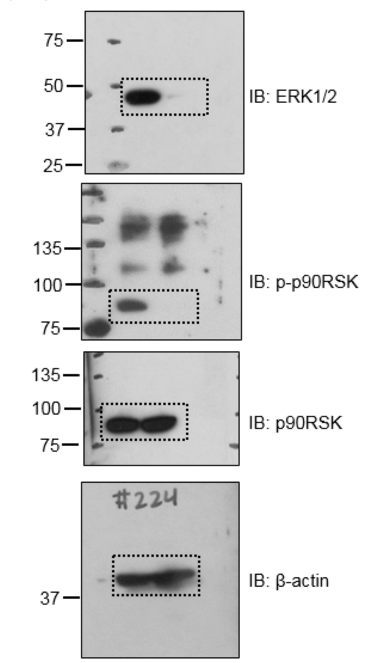

Fig. 6c

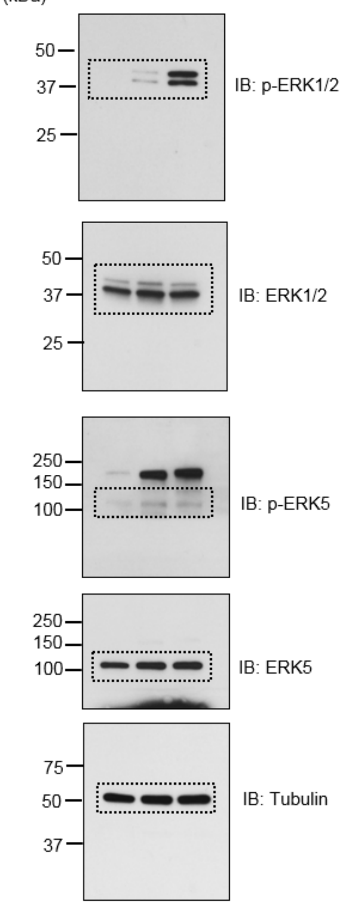

Supplementary Fig. 1b

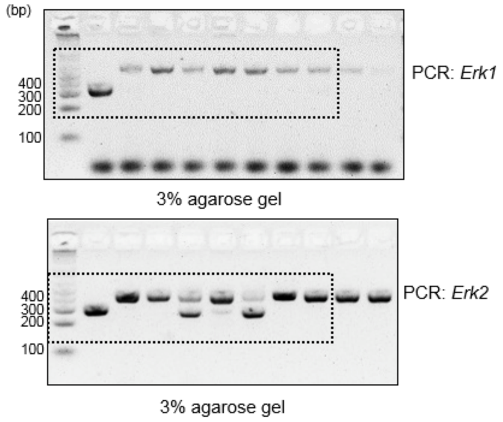

Supplementary Fig. 1c

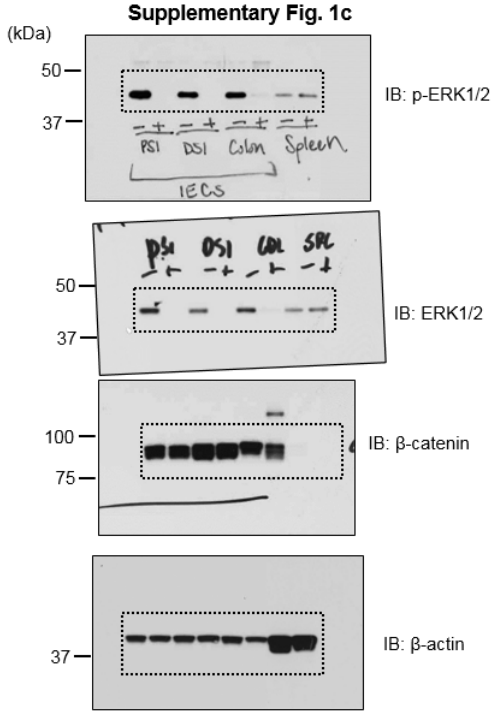

Supplementary Fig. 5d

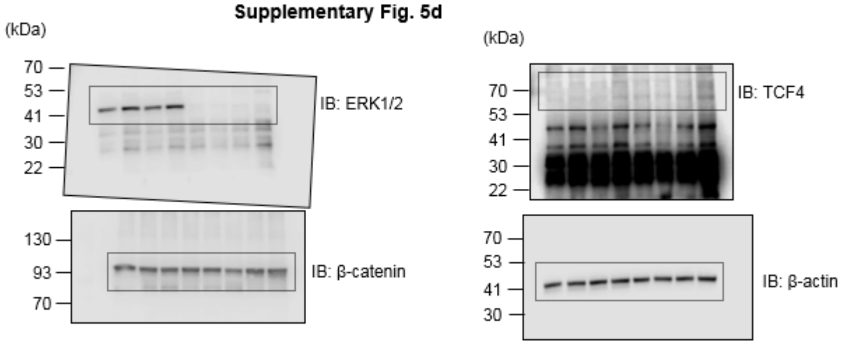

Supplementary Fig. 5e

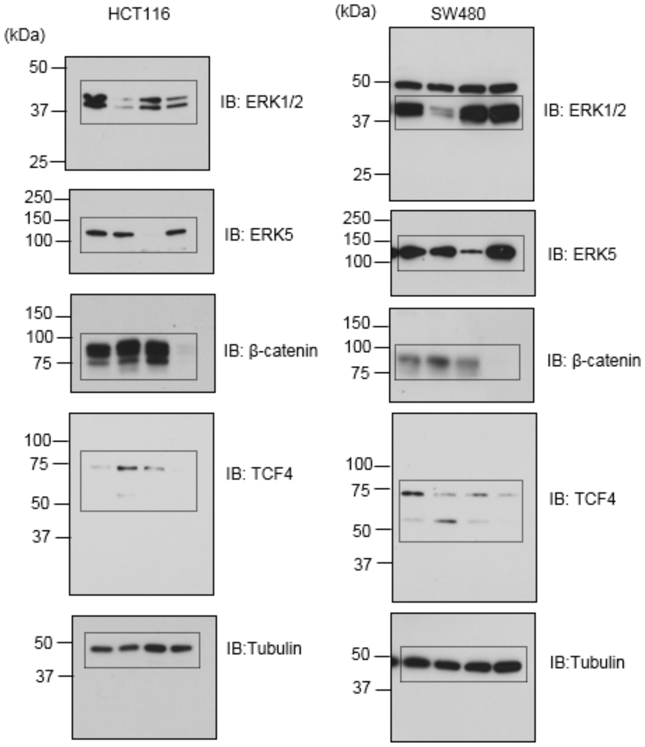

Supplementary Fig. 5h

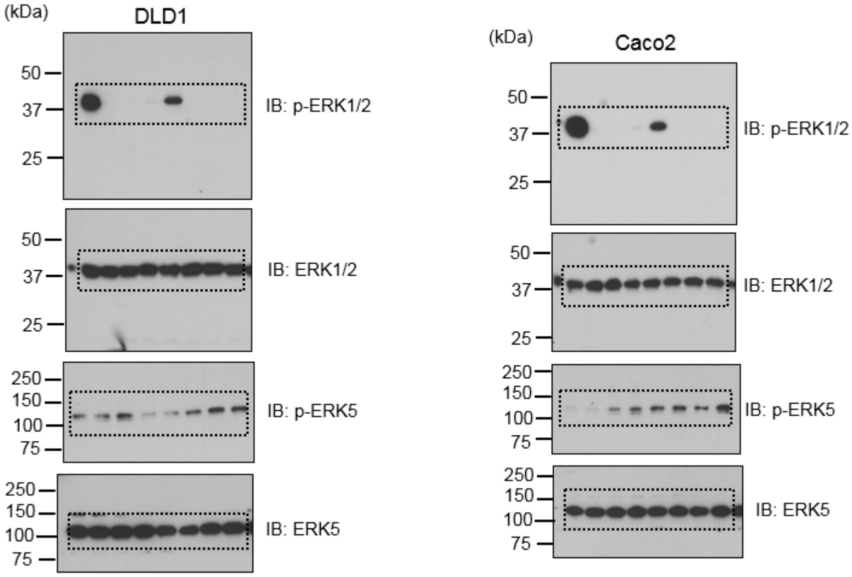

Supplementary Fig. 6b

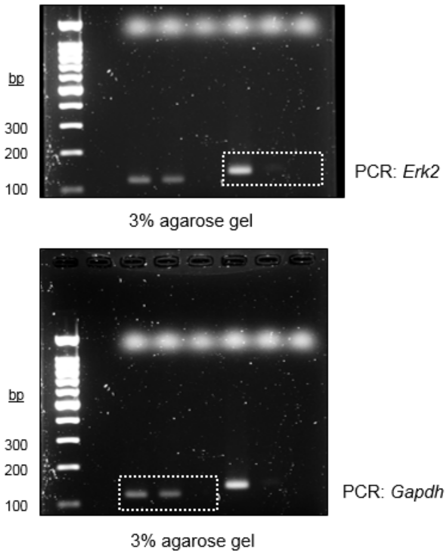

Supplementary Fig. 6g

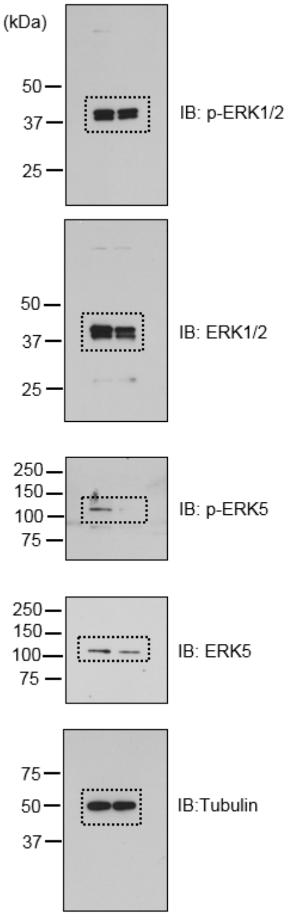

Supplementary Fig. 7i

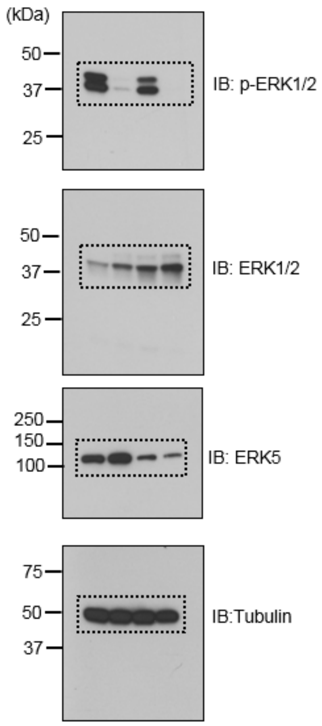

Supplementary Fig. 7l

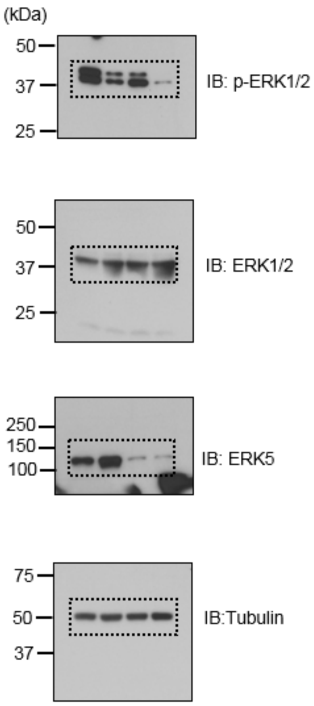

Supplementary Fig. 7j

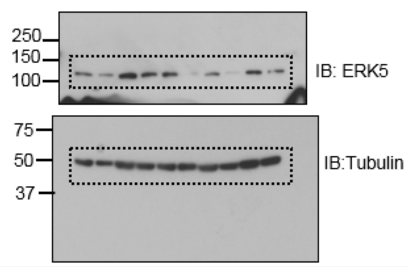

Supplementary Table 1. Primer sequences.

Gene	Accession number(s)	Sequence 5'-3'
18S rRNA	X00686.1	TTGACGGAAGGGCACCACCAG<br>GCACCACCACCCACGGAATCG
Ascl2	NM_008554	CCGGAGCATGGAAGCACACCTTG<br>AGGACTCCCTAGGGCACGCG
c-Fos	NM_010234.2	ACCATGATGTTCTCGGGTTTC<br>GCTGGTGGAGATGGCTGTCAC
c-Myc	NM_001177353.1<br>NM_010849.4	TGGTGTCTGTGGAGAAGAGGCAAA<br>TTGGCAGCTGGATAGTCCTTCCTT
Egr1	NM_007913	GTGTGCCCTCAGTAGCTTC<br>GACATCAATTGCATCTCGGC
Fra1 (Fosl1)	NM_010235.2	CCCTACCGAACATCCAGC<br>TTGGCACAAGGTGGAACT
Gapdh	NM_001289726.1<br>NM_008084.3	TCAACAGCAACTCCCACTCTT<br>ACCCTGTTGCTGTAGCCGTAT
Lgr5	NM_010195.2	TGAGCGGGACCTTGAAGATTTCCT<br>AGCCAGCTACCAAATAGGTGCTCA
Mapk1 (Erk2)	NM_011949.3<br>NM_001038663.1	TGCGCTTCAGACATGAGAAC<br>TGAGGTGCTGTGTCTTCAAG
Mapk7 (Erk5)	NM_011841.2<br>NM_001291033.1<br>NM_001291034.1<br>NM_001291035.1<br>NM_001291036.1	TCAAAGCTGCCCTGCTCAAG<br>TCTCTTCTCGTTCTCGCTGG
Mki67	NM_001081117.2	TGCCCGACCCTACAAAATG<br>GAGCCTGTATCACTCATCTGC
Mmp7	NM_010810.4	AACACTCTAGGTCATGCCTTCGCA<br>AGACCCAGAGAGTGGCCAAA
